# Total Body Irradiation Forever? Optimising Chemotherapeutic Options for Irradiation-Free Conditioning for Paediatric Acute Lymphoblastic Leukaemia

**DOI:** 10.3389/fped.2021.775485

**Published:** 2021-12-10

**Authors:** Khalil Ben Hassine, Madeleine Powys, Peter Svec, Miroslava Pozdechova, Birgitta Versluys, Marc Ansari, Peter J. Shaw

**Affiliations:** ^1^Cansearch Research Platform for Pediatric Oncology and Hematology, Department of Pediatrics, Gynecology and Obstetrics, Faculty of Medicine, University of Geneva, Geneva, Switzerland; ^2^Blood Transplant and Cell Therapies, Children's Hospital at Westmead, Sydney, NSW, Australia; ^3^Department of Pediatric Hematology and Oncology, Comenius University, Bratislava, Slovakia; ^4^Bone Marrow Transplantation Unit, National Institute of Children's Diseases, Bratislava, Slovakia; ^5^Princess Maxima Center for Pediatric Oncology, Utrecht, Netherlands; ^6^Division of Pediatric Oncology and Hematology, Department of Women, Child and Adolescent, University Geneva Hospitals, Geneva, Switzerland; ^7^Speciality of Child and Adolescent Health, Faculty of Medicine and Health, The University of Sydney, Sydney, NSW, Australia

**Keywords:** acute lymphoblastic leukaemia (ALL), hematopoietic stem cell transplant (HSCT), chemotherapy, pharmacokinetics, pharmacogenetics, pharmacodynamics (PD)

## Abstract

Total-body irradiation (TBI) based conditioning prior to allogeneic hematopoietic stem cell transplantation (HSCT) is generally regarded as the gold-standard for children >4 years of age with acute lymphoblastic leukaemia (ALL). Retrospective studies in the 1990's suggested better survival with irradiation, confirmed in a small randomised, prospective study in the early 2000's. Most recently, this was reconfirmed by the early results of the large, randomised, international, phase III FORUM study published in 2020. But we know survivors will suffer a multitude of long-term sequelae after TBI, including second malignancies, neurocognitive, endocrine and cardiometabolic effects. The drive to avoid TBI directs us to continue optimising irradiation-free, myeloablative conditioning. In chemotherapy-based conditioning, the dominant myeloablative effect is provided by the alkylating agents, most commonly busulfan or treosulfan. Busulfan with cyclophosphamide is a long-established alternative to TBI-based conditioning in ALL patients. Substituting fludarabine for cyclophosphamide reduces toxicity, but may not be as effective, prompting the addition of a third agent, such as thiotepa, melphalan, and now clofarabine. For busulfan, it's wide pharmacokinetic (PK) variability and narrow therapeutic window is well-known, with widespread use of therapeutic drug monitoring (TDM) to individualise dosing and control the cumulative busulfan exposure. The development of first-dose selection algorithms has helped achieve early, accurate busulfan levels within the targeted therapeutic window. In the future, predictive genetic variants, associated with differing busulfan exposures and toxicities, could be employed to further tailor individualised busulfan-based conditioning for ALL patients. Treosulfan-based conditioning leads to comparable outcomes to busulfan-based conditioning in paediatric ALL, without the need for TDM to date. Future PK evaluation and modelling may optimise therapy and improve outcome. More recently, the addition of clofarabine to busulfan/fludarabine has shown encouraging results when compared to TBI-based regimens. The combination shows activity in ALL as well as AML and deserves further evaluation. Like busulfan, optimization of chemotherapy conditioning may be enhanced by understanding not just the PK of clofarabine, fludarabine, treosulfan and other agents, but also the pharmacodynamics and pharmacogenetics, ideally in the context of a single disease such as ALL.

## The Evolution of HSCT Conditioning for Paediatric ALL

Total body irradiation (TBI)-based conditioning prior to allogeneic haemopoietic stem cell transplantation (HSCT) is generally regarded as the gold standard for children ≥4 years of age with acute lymphoblastic leukaemia (ALL). TBI is a powerful anti-leukaemic modality that eradicates leukaemia in sanctuary sites and reduces the risk of relapse post-transplant (1, 2). Unfortunately, survivors suffer a multitude of long-term sequelae after TBI including second malignancies and neurocognitive, endocrine and cardiometabolic effects (3). TBI also requires access to irradiation facilities and sedation or anaesthetic in young children. The drive to avoid TBI has inspired an international effort to develop irradiation-free myeloablative conditioning regimens that provide equivalent disease-free survival (DFS) to TBI without the associated toxicity for children requiring HSCT for ALL. This review outlines the evolution of TBI-based conditioning for paediatric ALL, the development of chemotherapy-based conditioning (chemo-conditioning) alternatives that culminated in the For Omitting Radiation Under Majority age (FORUM) trial, and the latest published myeloablative chemo-conditioning protocols for ALL.

### The Early Days of Chemo-Conditioning to Replace TBI

TBI conditioning prior to HSCT was pioneered by Thomas et al. in Seattle in 1970 (4). They added high-dose cyclophosphamide (120 mg/kg given over 2 days) to TBI in an effort to increase cytoreduction pre transplant and reduce relapse risk post-transplant. In a seminal report, they described the first 100 adult and paediatric patients with relapsed acute leukaemia who were transplanted in 1971–1975 following TBI-based conditioning (5). The combination of TBI and Cyclophosphamide was well-tolerated and was associated with long-term remission in 13% of patients, which was sustained in 8% (6). These results suggested that TBI-based conditioning for HSCT offered a survival advantage over chemotherapy in patients with end-stage disease, which prompted this approach to be trialled in the late 1970's in adult and paediatric patients with less-advanced leukaemia (7).

In the 1980's, attempts began to develop effective conditioning regimens that did not contain TBI, led by the John Hopkins group in Baltimore (8). They added the alkylating agent Busulfan to Cyclophosphamide to create the first chemo-conditioning regimen to be trialled. The addition of Busulfan aimed to provide equivalent myeloablation and leukaemia-free survival to TBI conditioning but with reduced toxicity. Chemo-conditioning with Busulfan 16 mg/kg and Cyclophosphamide 200 mg/kg or 120 mg/kg were used; both regimens induced long-term remission but the lower toxicity associated with Busulfan and Cyclophosphamide 120 mg/kg came at the cost of potentially increased relapse risk (9, 10). In paediatric HSCT, Busulfan and Cyclophosphamide 200 mg/kg is generally well-tolerated and so continues to be preferred over Busulfan and Cyclophosphamide 120 mg/kg as a conditioning regimen.

### Early Trials Comparing TBI With Busulfan Plus Cyclophosphamide Predominantly in Adults

In the early 1990's, the first four prospective, randomised controlled trials comparing TBI-based conditioning and chemo-conditioning were published by groups in France (11, 12), Scandinavia (13), and Seattle (14). The studies involved predominantly adult patients, although a small number of children were included. The most common indication for HSCT was myeloid disease [acute myeloid leukaemia [AML] or chronic myeloid leukaemia (CML)]; a minority of patients in the Scandinavian trial had ALL or lymphoma (13). In all four trials, patients received Cyclophosphamide 120 mg/kg. Those randomised to the chemo-conditioning received Busulfan 16 mg/kg. In the TBI arms, regimens varied with most receiving 12 Gy in fractionated doses. When first published, at a relatively short follow-up of 24–42 months, DFS was superior in patients that received TBI-based conditioning vs. chemo-conditioning for AML in CR1 in the French multicentre study (72 vs. 47%, *p* < 0.01) (11) and for adults with advanced myeloid or lymphoid disease in the Scandinavian randomised controlled trial (68 vs. 54%, *p* = 0.05) (13). In contrast, chemo-conditioning with Busulfan-Cyclophosphamide achieved equivalent DFS to TBI-based conditioning in patients with CML in results published by the Seattle (14) and French group (12). A subsequent meta-analysis of these studies, and an additional randomised controlled trial comparing conditioning with Busulfan-Cyclophosphamide against that with TBI and etoposide, confirmed a non-statistically significant trend toward better overall survival (OS) and DFS with TBI-based conditioning (15).

This trend favouring TBI over chemo-conditioning, particularly in AML, was supported by the publication of the long-term data of the four trials. At a median follow-up of 10.8 years, Blaise et al. continued to show that TBI-Cyclophosphamide was associated with statistically significant higher DFS and OS and decreased relapse rates and transplant-related mortality compared with conditioning with Busulfan-Cyclophosphamide in patients with AML (TBI-Cyclophosphamide: 10-year OS 59%, DFS 55%; Busulfan-Cyclophosphamide: 10-year OS 43%, DFS 35%) (16). In the update of the Scandinavian study at 7 years of follow-up, OS was also higher in the TBI group (63% with TBI-Cyclophosphamide vs. 54% with Busulfan-Cyclophosphamide group) but this difference was not statistically significant (17). Similarly, when Socie et al. combined the data from the original four trials, a non-statistically significant 10% lower OS was observed in patients with AML who received conditioning with Busulfan-Cyclophosphamide compared with in those who received TBI-Cyclophosphamide [projected 10-year survival: 51% for Busulfan-Cyclophosphamide vs. 63% for TBI-Cyclophosphamide, 95% confidence interval (CI) 52–74%]. No statistically significant difference in OS or DFS was observed among patients with CML, as in the original studies (18).

### Studies Comparing TBI With Busulfan Plus Cyclophosphamide in Children

In 2000, Davies et al. published a large study conducted in paediatric patients comparing TBI-based and chemo-conditioning regimens. This retrospective International Bone Marrow Transplant Registry (IBMTR) analysis included children with ALL who received a matched sibling HSCT after TBI/Cyclophosphamide or oral Busulfan-Cyclophosphamide. The incidence of relapse was similar between arms, suggesting that chemo-conditioning with Busulfan may not be inferior to TBI in preventing relapse. However, the higher non-relapse mortality (NRM) in the Busulfan arm led to TBI-based conditioning being associated with a superior leukaemia-free survival over Busulfan-based conditioning (50 vs. 35%, respectively; *p* = 0.005) (19).

The IBMTR study was shortly followed by publication of the first randomised controlled trial in paediatric patients comparing TBI-based and chemo-conditioning regimens: the Paediatric Blood and Marrow Transplant Consortium (PBMTC) study (20). This small study compared outcomes with chemo-conditioning with Busulfan, etoposide, Cyclophosphamide and anti-thymocyte globulin to those with TBI-based conditioning including Cyclophosphamide, etoposide +/- anti-thymocyte globulin. Relapse rates were similar between groups, yet NRM rates were higher in the Busulfan-Cyclophosphamide group. Bunin et al. concluded that “significant concerns regarding late effects, particularly secondary cancers, continue to make conditioning without radiation a potential attractive option, but additional studies are required to develop a safe, effective regimen.”

Despite these data, many centres replaced TBI-based protocols with Busulfan-based conditioning, particularly for myeloid diseases. However, over the ensuing decade, TBI retained its central role in conditioning for ALL. This was reinforced by evidence within in the literature. For example, a study looking at patients with ALL in CR2 concluded that TBI followed by HSCT compared to chemotherapy alone reduced the rate of relapse for children with early first relapse (21).

At the same time, there was continued recognition of the long-term burden following TBI, including an increased risk of breast cancer (22) and thyroid cancer (23). Moreover, the association between an increased risk of second solid cancers and age at the time of TBI was reported (24).

An important point is that in all the above studies, the Busulfan preparation used was oral, not intravenous (IV). The highly variable absorption rate and bioavailability of Busulfan, adding to its variable clearance, led to the development of the IV Busulfan formulation (25, 26). IV Busulfan enables better control of the cumulative exposure to Busulfan through therapeutic drug monitoring (TDM) (27, 28). In a retrospective trial that included paediatric ALL patients, Bartelink et al. reported an improved event-free survival (EFS) (83 vs. 30%, respectively; *p* < 0.001) and OS (83 vs. 53%, respectively; *p* = 0.016) accompanied with a decrease risk of veno-occlusive disease (VOD) under TDM-guided IV Busulfan compared with fixed-dose oral Busulfan (27). Although most centres have moved to the IV route, oral administration of Busulfan in paediatric HSCT is still used. Of note, a retrospective registry-based study on 460 transplanted children with leukaemia showed similar outcomes for both IV and oral formulations of Busulfan, but it was suggested that this was likely due to the routine use of Busulfan TDM (29).

### The FORUM Trial of TBI vs. Chemo-Conditioning

With recognition of the life-long consequences of irradiation in young children, a convergence of shared thoughts and ideas led to the creation of the protocol that became the FORUM international, randomised controlled trial (Clinicaltrials.gov identifier: NCT02670564). The rationale included the following points:

1. Some patients relapse after TBI-based conditioning.
2. The use of oral Busulfan was being replaced by IV Busulfan, supporting more consistent bioavailability, more predictable pharmacokinetics (PK) and lower incidence of acute toxicity.
3. Recognition of the importance of measurable residual disease (MRD), particularly at the time of HSCT, for identifying patients with a poorer prognosis even with TBI-based HSCT (30, 31).
4. The use of haploidentical donors for second or third, and more recently first, HSCT was increasing; these transplants had often used less-aggressive conditioning than first or second remission transplants using matched related or unrelated donors. Despite the less intensive conditioning, the good overall results suggested that the greater immune reactivity of the mismatched donor might favour a graft-versus-leukaemia effect (32, 33).
5. A non-significant trend in favour of disease control by TBI in early follow-up might be offset in later follow-up by benefits of chemo-conditioning in terms of hard endpoints such as rates of secondary malignancies and other multiple benefits, such as a reduced risk of cataracts as well as fewer growth, neurocognition and dental effects.

The FORUM trial compared TBI (12 Gy) plus etoposide vs. chemo-conditioning with Fludarabine and Thiotepa combined with either Busulfan or Treosulfan (by country preference) in paediatric patients with ALL in CR who were between the ages of 4 and 21 years at HSCT. Twenty-one countries were involved in this large, prospective, Phase III study. The original intention was to recruit 1,000 patients over 5 years; however, the trial was stopped in March 2019 after 417 patients had been randomised due to early results indicating superiority of the TBI arm. The early results of FORUM were published in 2021 and confirmed that TBI conditioning was superior to chemo-conditioning, with a 16% higher 2-year OS (91 vs. 75%, respectively; *p* < 0.0001) and reduced cumulative risk of relapse (12 vs. 33%, respectively; *p* < 0.0001). Treatment-related mortality (TRM) was similar between the groups (34).

With FORUM showing a clear early benefit favouring TBI, we have to rethink how conditioning therapy in childhood ALL might otherwise be improved. Options include:

- Optimising the use of Busulfan-based conditioning with PK and genomics
- Optimising the use of Treosulfan
- Optimising the whole conditioning regimen
- Introducing newer agents, such as clofarabine (Clo), into conditioning regimens and establishing how we can introduce a new combination into frontline HSCT therapy.

We now explore each of these themes in turn.

## Optimising the Use of Busulfan-Based Conditioning With Pharmacokinetics and Genomics

### Definition and Refinement of the Optimal Busulfan Target Exposure

Busulfan with TDM is recommended in paediatric HSCT for several reasons. Firstly, Busulfan has a demonstrated exposure-response relationships and narrow therapeutic window, so small variations in exposure can result in poor clinical outcomes. Secondly, despite the improved predictability of PK obtained using IV formulations, due to the bypass of the unpredictable absorption phase, the inter-individual and intra-individual PK variability in Busulfan elimination and exposure remain substantial. The American Society for Blood and Marrow Transplantation recommends TDM-based dose adjustments for paediatric patients receiving myeloablative Busulfan-based conditioning therapy (35).

The association between Busulfan exposure and outcomes in paediatric patients with varying malignant diagnoses, including ALL, has been reported in many studies (Table 1) (36–55). The therapeutic window for Busulfan recommended by the European Medicines Agency (EMA) is AUC_6h_ 900–1,500 μM.min (daily AUC of 14.8–24.6 mg.h/L) (56, 57). This target was originally derived from studies in adult HSCT patients using oral Busulfan. Exposure higher than 1,500 μM.min has been associated with increased toxicities such as sinusoidal obstruction syndrome (SOS) and acute graft-versus-host disease (GvHD) (47, 58, 59), while exposures lower than 900 μM.min were associated with increased graft rejection and disease relapse (52, 60). This therapeutic window has been confirmed to be safe and efficacious in various studies of paediatric patients, including those with ALL (52, 61, 62). Nguyen et al. developed a dosing nomogram designed to reach this therapeutic target in paediatric patients, which the EMA has since recommended (57). One retrospective study in 138 patients, including 13 paediatric patients with ALL, investigated the impact of narrowing the EMA-recommended typical Busulfan therapeutic window to a local target AUC_6h_ 980–1,250 μM.min (daily AUC 16.1–20.5 mg.h/L). The efficacy (EFS and OS) and safety (SOS) outcomes evaluated in this study cohort were not improved using a narrower therapeutic window, suggesting that the EMA therapeutic window of 900–1,500 μM.min (daily AUC of 14.8–24.6 mg.h/L) is the most appropriate for children (53).

**Table 1:** Summary of studies assessing exposure response to busulfan.

**References**	**Population**	**Conditioning regimen**	**TDM dose adjustment?**	**Tested outcome**	**Exposure-response result**	**Other covariates influencing the outcome**
Bartelink et al. (36)	*N* = 674 Age range: – 30.4 (median 4.5) Haematological malignancies: 41%	IV Q6 h and Q24 h BuCy (52%) BuFlu (38%) BuCyMel (10%)	Yes, target defined by the treatment centres	EFS	AUC_cum_ < 78 mg.h/L: 66.1% EFS at 2 years vs. AUC_cum_ < 78 mg.h/L: AUC_cum_ 78−101 mg.h/L: 81% EFS at 2 years HR = 0.64, p =0.004 AUC_cum_ >101 mg.h/L: 49.5% EFS at 2 years, HR = 1.21, NS	Immunodeficiency diagnoses vs. other non-malignant diseases
				OS	Vs. AUC_cum_ < 78 mg.h/L: AUC_cum_ 78−101 mg.h/L: HR = 0.53, *p* = 0.016 AUC_cum_ >101 mg.h/L: HR = 1.03, NS	
				Graft failure/relapse	Vs. AUC_cum_ < 78 mg.h/L: AUC_cum_ 78−101 mg.h/L: HR = 0.57, *p* = 0.004 AUC_cum_ >101 mg.h/L: HR = 0.41, *p* = 0.094	
				TRM	Vs. AUC_cum_ < 78 mg.h/L: AUC_cum_ 78−101 mg.h/L: HR = 1.07, NS AUC_cum_ >101 mg.h/L: HR = 2.99, *p* < 0.001	Use of three alkylating agents
				Acute toxicity: SOS grade II–IV and aGvHD grade II–IV	Vs. AUC_cum_ < 78mg.h/L: AUC_cum_ 78−101 mg.h/L: HR = 1.14, *p* = NS AUC_cum_ >101 mg.h/L: HR = 1.69, *p* = 0.013	Use of three alkylating agents, transplant after 2006
				cGvHD	AUC_cum_ < 78 mg.h/L: 4.3% cGvHD AUC_cum_ >78 mg.h/L: HR = 1.3, NS	
				cGvHD-free, event-free survival	Vs. AUC_cum_ < 78 mg.h/L: AUC_cum_ 78−101 mg.h/L: HR = 0.57, *p* < 0.001 AUC_cum_ >101 mg.h/L: HR = 1.38, NS	
Bartelink et al. (37)	*N* = 102 Age range: 0.1–21.0 years (median 3.1) Haematological malignancies: 46%	IV q6h and q24h BuCyMel (43%) Others (57%): Bu combined with Cy, Flu or/and VP16	Yes, three different AUC_cum_ targets: 78.8 mg.h/L 62.4 mg.h/L 70.0 mg.h/L	EFS	AUC_cum_ 72–80 mg.h/L: highest EFS (*p* = 0.028) Optimal AUC_cum_: 74–82 mg.h/L	HLA disparity, age
				OS	AUC_cum_ 72–80 mg.h/L: highest OS (*p* = 0.021)	HLA disparity, age
				Graft failure/relapse	AUC_cum_ >72.5 mg.h/L: HR = 0.47, *p* = 0.004 vs. AUC_cum_ < 72.5 mg.h/L	
				SOS (grade II–IV)	In patients given BuCyMel: AUC_cum_ >74 mg.h/L: HR = 4.1, *p* = 0.012 vs. AUC_cum_ < 74mg.h/L	Mel-containing regimens
				aGvHD (grade II–IV)	AUC_cum_ is a significant predictor of aGvHD (HR = 1.56; *p* = 0.019) In patients given BuCyMel: AUC_cum_ >74 mg.h/L: HR = 4.5, *p* = 0.016 vs. AUC_cum_ < 74 mg.h/L	Mel-containing regimens
				Mucositis	NS	Mel-containing regimens
				Acute lung toxicity	NS	
Ansari et al. (38)	*N* = 75 Age range: 0.1–20 years (median 6.2) Haematological malignancies: 64%	IV q6 h BuCy (89%) BuCyVP16 (8%) BuMel (3%)	Yes, from the 5th dose for a target Css of 600–900 ng/mL (AUC_cum_ 57.6–86.4 mg.h/L)	EFS	First dose Css >600 ng/mL (AUC_6h_ >3.6 mg.h/L): higher event incidence, HR=5.14, *p* < 0.001 vs. Css <600 ng/ml	
				OS	First dose Css >600 ng/mL (AUC_6h_ >3.6 mg.h/L): higher mortality, HR = 7.55, *p* = 0.001 vs. Css <600 ng/ml	
				NRM	First dose Css >600 ng/mL (AUC_6h_ >3.6 mg.h/L): higher NRM, HR = 7.55, *p* = 0.001 vs. Css <600 ng/ml	
				Relapse	First dose Css >600 ng/mL (AUC_6h_ >3.6 mg.h/L): tendency of higher incidence of relapse (41 vs. 23%, *p* = 0.13) vs. Css <600 ng/ml	
				aGvHD (grade II–IV)	First dose Css >600 ng/mL (AUC_6h_ >3.6 mg.h/L): higher incidence of aGVHD (21 vs. 5%, *p* = 0.04) vs. Css <600 ng/ml	
				SOS	First dose Css >600 ng/mL (AUC_6h_ >3.6 mg.h/L): tendency of higher incidence of SOS (*p* = 0.12) vs. Css <600 ng/ml	
				Lung toxicity	First dose Css >600 ng/mL (AUC_6h_ >3.6 mg.h/L): tendency of higher incidence of lung toxicity (*p* = 0.06) vs. Css <600 ng/ml	
				Haemorrhagic cystitis	First dose Css >600 ng/mL (AUC_6h_ >3.6 mg.h/L): tendency of higher incidence of HC (*p* = 0.07) vs. Css <600 ng/ml	
Ansari et al. (39)	*N* = 108 Age range: 0.1–19.9 years (median 5.8) Haematological malignancies: 64%	IV q6 h BuCy (76.8%) BuCyVP16 (10.9%) BuMel (1.4%) BuCyMel (10.9%)	Yes, target defined by the treatment centres	EFS	First dose Css <600 ng/mL (AUC_6h_ < 3.6 mg.h/L): event incidence of 17% First dose Css 600–900 ng/mL (AUC_6h_ 3.6–5.4 mg.h/L): event incidence of 50% First dose Css >900 ng/mL (AUC_6h_ > 5.4 mg.h/L): event incidence of 65% *p* < 0.001	
				OS	First dose Css <600 ng/mL (AUC_6h_ < 3.6 mg.h/L): event incidence of 7% First dose Css 600–900 ng/mL (AUC_6h_ 3.6–5.4 mg.h/L): event incidence of 38% First dose Css >900 ng/mL (AUC_6h_ > 5.4 mg.h/L): event incidence of 60% *p* < 0.001	*GSTA1* polymorphisms
				TRT	First dose Css <600 ng/mL (AUC_6h_ < 3.6 mg.h/L): event incidence of 40% First dose Css 600–900 ng/mL (AUC_6h_ 3.6–5.4 mg.h/L): event incidence of 48% First dose Css >900 ng/mL (AUC_6h_ >5.4 mg.h/L): event incidence of 85% *p* < 0.001 First dose Css >900 ng/mL: significantly higher TRT in GSTA1-slow-metabolising patients (88 vs. 37%, *p* < 0.0005)	*GSTA1* polymorphisms
Baker et al. (40)	*N* = 52 Age range: 0.1–53 years (median 9.2) Haematological malignancies: 100% (AML)	Oral q6h Bu with Cy	No	Relapse	NS	
				OS	First dose Css <578 ng/mL (AUC_6h_ < 3.5 mg.h/L): trend of improved OS (69 vs. 49% at 3 years, *p* = 0.07) vs. Css >578 ng/ml	
				DFS	First dose Css <578 ng/mL (AUC_6h_ < 3.5 mg.h/L): improved DFS (63 vs. 42% at 3 years, *p* = 0.05) vs. Css >578 ng/ml	
				NRM	First dose Css >578 ng/mL (AUC_6h_ >3.5 mg.h/L): higher risk of NRM (30 vs. 8% at 3 years, *p* = 0.06) vs. Css >578 ng/ml	
				aGvHD	NS	
Bartelink et al. (41)	*N* = 674 Age range: 0.1–30.4 years (median 4.5) Haematological malignancies: 41%	IV q6 h and q24 h BuCy (52%) BuFlu (38%) BuCyMel (10%)	Yes, target defined by the treatment centres	EFS	AUC_cum_ 78–101 mg.h/L vs. AUC_cum_ 59–99 mg.h/L (EMA): HR = 0.91, *p* = NS AUC_cum_ 78–101 mg.h/L vs. AUC_cum_ 59–89 mg.h/L (FDA): HR = 0.66, *p* = 0.024 AUC_cum_ 78–101 mg.h/L vs. AUC_cum_ 59–78 mg.h/L: HR = 0.78, *p* = 0.035	
Benadiba et al. (42)	*N* = 36 cord blood transplanted patients Age range: 0.6–19.3 years (median 5.9) Haematological malignancies: 100% (AML or MDS)	IV q6 h BuCy (91.7%) BuCyVP16 (5.6%) BuMel (2.8%)HC	Yes, from the 5th dose for a target Css of 600–900 ng/mL (AUC_cum_ 57.6–86.4 mg.h/L)	EFS	First dose Css >600 ng/mL (AUC_6h_ >3.7 mg.h/L): higher incidence of event, HR = 3.83, *p* = 0.01 vs. Css <600 ng/ml	
				OS	First dose Css >600 ng/mL (AUC_6h_ >3.7 mg.h/L): higher mortality, HR = 5.2, *p* = 0.02 vs. Css <600 ng/ml	
				NRM	First dose Css >600 ng/mL (AUC_6h_ >3.7 mg.h/L): higher NRM (28.6 vs. 0%, *p* = 0.009) vs. Css <600 ng/ml	
				Neutrophil recovery	First dose Css >600 ng/mL (AUC_6h_ >3.7 mg.h/L): lower neutrophil recovery incidence (95.5 vs. 75.5%, *p* = 0.01) vs. Css <600 ng/ml	
				Platelet recovery	First dose Css >600 ng/mL (AUC_6h_ >3.7 mg.h/L): lower platelet recovery incidence (67.9 vs. 100%, *p* = 0.04) vs. Css <600 ng/ml	
				SOS	NS	
				aGvHD grade II–IV	NS	
				Lung-toxicity	NS	
				Hemorrhagic cystitis	First dose Css >600 ng/mL (AUC_6h_ >3.7 mg.h/L): higher HC incidence (50.0 vs. 18%, *p* = 0.04) vs. Css <600 ng/ml	
				Relapse	NS	MDS, cord blood compatibility (trends)
Bolinger et al. (43)	*N* = 38 Age range: 0.6–18 years Haematological malignancies: 37% (AML)	Oral q6 h Bu followed by Cy	No	Graft rejection TRT	First dose Css >600 ng/mL (daily AUC <14.4 mg.h/L): lower incidence of graft rejection (0 vs. 35%, *p* = 0.018) vs. Css <600 ng/ml NS	
Bolinger et al. (44)	*N* = 39 Age range: 0.6–18.5 years Haematological malignancies: 41% (23% AML)	Oral q6 h Bu followed by Cy	Yes, following a test dose, and at dose 5, 9, and/or 13 if necessary to a Css range of 600–900 ng/ml ± 10% (AUC_cum_ 57.6 – 86.4 mg.h/L ± 10%)	Graft rejection	Overall Css 600–900 ng/mL (daily AUC 14.4 – 21.6 mg.h/L): higher rate of engraftment (94 vs. 74%, *p* = 0.043) vs. Css <600 ng/ml	
				TRT	Trend of increased grade III–IV TRT with increasing Bu overall CSS	
Copelan et al. (45)	*N* = 28 Age range: 4–54 years (6 patients < 18 years) Haematological malignancies: 100%	Oral q6 h Bu followed by Cy	No	Early TRM (6 months post transplantation) SOS	Trend of early TRM associated with high first dose AUC_6h_ (*p* = 0.06) SOS significantly associated with high first dose AUC_6h_ (*p* = 0.03)	
				Relapse	NS	
				Late NRM	NS	
				EFS	NS	
				cGVHD	NS	
				Obstructive bronchiolitis	NS	
Esteves et al. (46)	*N* = 202 Age: 31% < 18 years Haematological malignancies: 81% (10% ALL)	IV q24 h Bu with other agents (Cy, Flu, Mel, and/or Thio) Oral q6h Bu followed by Cy	Yes, according to test dose PK. Three defined AUC_cum_ targets: 49.3 mg.h/L 65.7 mg.h/L 82.1 mg.h/L Historical control group: no TDM	SOS Oral mucositis Relapse EFS OS	Increased SOS with AUC_24h_ >5,000 μM.min (AUC_24h_ >20.5 mg.h/L (HR = 3.39, *p* = 0.034) vs. AUC_24h_ <5,000 μM.min NS NS NS NS	
Grochow et al. (47)	*N* = 30 Age range: NR Included paediatric patients and haematological malignancies.	Oral q6 h Bu followed by Cy	No	SOS	The incidence of SOS correlated with first dose AUC_6h_ >3,200 μM.min (AUC_6h_ >13.1 mg.h/L): (χ^2^ =18; *p* < 0.0001) vs. AUC_6h_ <3,200 μM.min	
Kerl et al. (48)	*N* = 59 Age range: 0.2–18.7 years Diagnoses non-reported	IV q6 h or q24 h Bu followed by Cy	Only in q24 h patients	SOS	The incidence of SOS correlated with higher first dose AUC only in q6h patients (*p* < 0.05)	
Ljungman et al. (49)	*N* = 172 Age range: 1.2–65 years (median 36) Haematological malignancies: 100%	Oral q6 h Bu followed by Cy	No	TRM	Bu concentration ≥721 ng/mL: increased TRM during the 1st year after transplantation (29 vs. 14%, *p* = 0.01) vs. Css <721 ng/ml	
				OS	Bu concentration ≥721 ng/mL: decreased OS (56 vs. 40%, *p* = 0.05) vs. Css <721 ng/ml Autologous HSCT only: NS	
				DFS	Bu concentration ≥721 ng/mL: decreased DFS(51 vs. 37%, *p* = 0.03) vs. Css <721 ng/ml Autologous HSCT only: NS	
				Relapse	NS	
Philippe et al. (50)	*N* = 293 Age range: 0.2–21 years (mean 6.5) Haematological malignancies: 42.7% (1 ALL patient)	IV q6h, q12h, and q24h Bu with Cy, Flu, Mel, Thio, or/and VP16	Yes, to target an AUC_6h_ of 900–1,500 μM.min (3.7–6.1 mg h/L)	SOS	Univariate analysis: first dose AUC, Cmax, percentage of time above 1,300 ng/mL associated with SOS. Multivariate analysis: highest Cmax associated with SOS	Age <3 years, weight <9 kg, severe combined immunodeficiency or a lymphohistiocytosis, VP16
				Engraftment	AUC_cum_ associated with engraftment	Weight, age, haematological malignant disease, Cy co-administration associated with engraftment Flu co-administration associated with rejection
Zwaveling et al. (51)	*N* = 31 Age range: 0.22–14 (median 5.0) Haematological malignancies: 58%	IV q6h Bu BuCy (35%) BuCyMel (48%) BuCyVP16 (6%) FluBuCy (10%)	Yes, from the 2nd day of treatment	SOS OS Engraftment Relapse	No association between AUC_cum_ and SOS No association between AUC_cum_ and OS No association between AUC_cum_ and engraftment No association between AUC_cum_ and relapse	
McCune et al. (52)	*N* = 53 Age range: 1.2 - 65 (median 36) Haematological malignancies: 55% (1 ALL patient)	Oral q6 h Bu followed by Cy	From the 2nd day of treatment	Graft rejection TRT	Risk of rejection decreasing with increased Css (*P* = 0.0024) Severe TRT were not related to Css	
Philippe et al. (53)	*N* = 138 Age range: 0.17 – 21 (median 5) Haematological malignancies: 50.7% (13 ALL patients)	IV q6h Bu with Cy, Flu, Mel, Thio, or/and VP16	Yes, to target an AUC_6h_ of 980–1,250 μM.min (4.0 – 5.1 mg.h/L)	SOS-free survival at 1 month post HSCT SOS	No difference between patients within a local AUC range (AUC_6h_ 4.0 – 5.1 mg.h/L) and the EMA AUC range (AUC_6h_ 3.7 – 6.2 mg.h/L) No correlation between first dose AUC and cumulative AUC with SOS. No difference between patients within a local AUC range (AUC6 h 4.0 – 5.1 mg.h/L) and the EMA AUC range (AUC6 h 3.7 – 6.2 mg.h/L)	Patients < 9 kg
				Engraftment	No correlation between first dose AUC and cumulative AUC with SOS.	Non-malignancies
				OS	No difference between patients within a local AUC range (AUC_6h_ 4.0 – 5.1 mg.h/L) and the EMA AUC range (AUC_6h_ 3.7 – 6.2 mg.h/L)	
				Relapse	higher probability with AUCcum <3.7 mg.h/L, 42.9%) than in patients within EMA target range (AUC_6h_ 3.7 – 6.2 mg.h/L)	
Schechter et al. (54)	*N* = 47 Age range: 0.25 – 16.2 (median 5.1) Haematological malignancies: 29.7% (No ALL patients)	IV q6 h Bu with Cy, Mel, Thio or/and VP16	Yes, to target an AUC_6h_ of 900–1,500 μM.min (3.7–6.1 mg h/L)	SOS	Higher Cmax in patients who developed SOS (4.2 ± 0.68 vs. 4.8 ± 0.73 μM; *P* = 0.035)	
Bouligand et al. (55)	*N* = 45 Age range: 1.2 – 20 (median 5.1) 1 Lymphoma patient. Mainly neuroblastoma, medulloblastoma or Ewing sarcoma diagnoses	Oral q6 h Bu with either Mel or Thio	No	SOS	BuThio patients with SOS had a significantly higher AUC_6h_ after the 13th dose (6.201 ± 0.607 mg.h/L) than those who did not (5.024 ± 0.978 mg.h/L) (*P* < 0.05) This difference was not observed in patients that received BuMel	Second alkylating agent: Mel or Thio

*aGvHD, acute graft-versus-host disease; AML, acute lymphoblastic leukaemia; AUC, area under the curve; Bu, busulfan; cGvHD, chronic graft-versus-host disease; Css, steady state concentration; Cy, cyclophosphamide; DFS, disease-free survival; EFS, event-free-survival; EMA, European Medicines Agency; FDA, US Food and Drug Administration; Flu, fludarabine; GSTA1, glutathione S-transferase A1; HC, haemorrhagic cystitis; HLA, human leukocyte antigen; HR, hazard ratio; IV, intravenous; MDS, myelodysplastic syndrome; Mel, melphalan; NRM, Non-relapse mortality; NS, not significant; OS, overall survival; q24h, every 24 hours; q6h, every 6 hours; SOS, sinusoidal obstruction syndrome; TDM, therapeutic drug monitoring; Thio, thiotepa; TRM, treatment-related mortality; TRT, treatment-related toxicity; VP16, etoposide*.

Another target for Busulfan dosing is based on steady-state concentration (Css). Css values can be expressed as AUC values by multiplying the Css value by the inter-dose interval. The reported optimal Css window of Busulfan is 600–900 ng/mL, corresponding to a daily AUC of 14.4 −21.6 mg.h/L (43, 44), which is only slightly lower than another narrowed therapeutic window recommended by the US Food and Drug Administration (FDA) (daily AUC 14.8–22.2 mg.h/L) (63). A recent meta-analysis by Feng et al. showed that the typical lower cutoff of 900 μM.min (daily AUC 14.8 mg.h/L) was strongly associated with the risk of graft failure (AUC ≥900 μM.min vs. <900 μM.min: Relative risk (RR) 3.666; CI 1.419–9.467), while the FDA cutoff (1,350 μM.min; daily AUC 22.2 mg.h/L) was more strongly associated with the risk of SOS than the EMA target (AUC ≤ 1,350 μM.min vs. >1,350 μM.min: RR 0.370; CI 0.205–0.666) (64). This study suggested that the FDA upper AUC cutoff (1,350 μM.min, daily AUC 22.2 mg.h/L) is safer in paediatric patients in terms of protection from SOS.

Much of the discussion about the Busulfan exposure metric has been superseded with the international harmonisation process to adopt uniform units of mg.L.h (65), as used in the largest retrospective study to date on the association between Busulfan exposure and outcomes in paediatric patients (36). Of the 674 patients enrolled in that study by Bartelink and colleagues, 41% were diagnosed with malignancies but only 5% had ALL (36). Based on EFS as the main criteria, the researchers found the optimal therapeutic window to be 78–101 mg.h/L, corresponding to a daily AUC of 19.5–25.3 mg.h/L. This target was shown to be optimal regardless of patients' malignant diagnoses. This new therapeutic target is included within the EMA target, with a slightly higher upper range (25.3 vs. 24.6 mg.h/L, respectively). However, it is higher than the FDA target, which was reported to be associated with a decreased SOS risk (64). This therapeutic window proposed by Bartelink et al. was also associated with acceptable acute toxicity (defined as acute GvHD and SOS) and occurrence of chronic GvHD. In response to a letter to the editor by Paci et al. (66), Bartelink et al. demonstrated that EFS was significantly reduced when targeting the lower end of the EMA threshold (AUC 59–78 mg.h/L) (41). The different studies show that there is still no consensus on the optimal cumulative exposure to Busulfan for paediatric patients due to heterogeneous data. Future well-designed, prospective investigations should further establish the optimal target window of Busulfan. However, it is widely agreed that TDM-guided dose adjustment of Busulfan is required to reach the desired target exposure in the paediatric HSCT setting, especially in neonates and small children for whom Busulfan PK is more unpredictable (67).

Studies have also shown that HSCT outcomes are not only associated with cumulative exposure to Busulfan but also with per-dose exposure. The AUC or Css of the first dose of Busulfan has been reported to be associated with toxicities of Busulfan as well as transplant outcomes. As shown in Table 1, a study from Ansari et al. reported that a first-dose Css <600 ng/mL (AUC_6h_ <3.6 mg.h/L) was associated with improved OS and EFS, a lower NRM and a lower incidence of relapse and acute GvHD of grade II to IV compared to patients with Css > 600 ng/mL (38). The other toxicities reported (SOS, lung toxicities, and haemorrhagic cystitis) showed trends of lower incidence in patients receiving Busulfan with a first-dose Css <600 ng/mL (AUC_6h_ <3.6 mg.h/L) compared to patients with Css > 600 ng/mL. A similar association between this exposure cut off and better NRM, OS, and EFS was later demonstrated in a larger multicentre population (39). In the latter study, the association between exposure and treatment-related toxicity (TRT) risk, comprising acute GvHD of grade I–IV, was shown to depend on glutathione S-transferase A1 (GSTA1) metabolic capacity (39).

Another study reported the association between SOS with the per-dose PK parameters of Busulfan in 293 patients including 125 with haematological malignancies (50). In the univariate analysis based on logistic regression, the maximal concentration after Busulfan infusion ended, and the first-dose AUC, but not the cumulative AUC, were associated with the occurrence of SOS. In the same study, engraftment only significantly associated with cumulative AUC. Interestingly, a study by Kerl et al. reported an increased risk of SOS with AUC_6h_ >1,500 μM.min (daily AUC >24.6 mg.h/L) in patients receiving Busulfan four times daily but not in patients receiving once daily Busulfan (48). These studies provide evidence that per-dose exposure to Busulfan could impact the outcomes and incidence of toxicity in paediatric patients. Accurately targeted first doses of Busulfan before TDM is performed should enable clinicians to avoid the toxicities and poor outcomes related to higher per-dose exposure. A planned future analysis of Busulfan PK data from the FORUM trial will enable better understanding of the association between Busulfan exposure and outcomes in a homogenous cohort of paediatric ALL patients. A similar analysis will be performed of Busulfan exposure in AML patients in the ongoing Myechild01 trial (Clinicaltrials.gov identifier: NCT02724163). The target Busulfan exposure in FORUM and TDM adjustment settings were not harmonised; rather, they depended on the local clinical practise in each transplantation centre (34). The upcoming analysis of the FORUM PK data will enable the researchers to explore a potentially heterogeneous Busulfan exposure among patients and its relationship to patient outcomes. This heterogeneity in patient exposure could partly explain the inferiority of Busulfan-based regimens to TBI, and the analysis of the Busulfan PK data from FORUM will explore this.

### Busulfan Administration Schedule

In HSCT, Busulfan was originally administered during 4 days of conditioning, four times daily (every 6 h). A once daily oral or IV Busulfan schedule has been reported to be safe and efficacious in paediatric patients (27, 68–72). One study in paediatric patients receiving IV Busulfan compared SOS risk between once-daily and four-times-daily dosing, finding a similar risk with each schedule (48). However, an association between exposure and SOS was only observed in patients receiving Busulfan four times a day, probably due to the presence of other risk factors. More recently, Philippe et al. showed that the risk of SOS was associated with the maximum concentration (C_max_) of Busulfan. While the cumulative AUC should be equivalent between once-daily and four-times-daily dosing, the C_max_ obtained with once-daily dosing is systematically higher than that obtained with four-times-daily dosing.

The study by Philippe et al. included 11 patients who received once-daily or twice-daily Busulfan, among which nine (81.8%) patients experienced SOS (50). In contrast, other studies in paediatric patients have observed a lower occurrence of SOS in paediatric patients who received once-daily IV Busulfan dosing (69, 70). Further studies should address the comparison between once-daily and four-times-daily IV Busulfan dosing in paediatric patients, in terms of efficacy and toxicity outcomes.

The once-daily Busulfan dosing schedule has many advantages. Xhaard et al. showed that once-daily Busulfan dosing was associated with better patient comfort related to reduced nausea and vomiting and less infusions (73). Once-daily dosing was perceived by healthcare professionals to be safer and less error prone, in addition to reducing workload and allowing smoother treatment management. In addition, once-daily Busulfan dosing reduces transplantation-related costs (74). Dividing the total Busulfan dose over 16 doses (four times a day schedule) provides more opportunity for dose adjustments, which may make it easier to target the desired cumulative exposure. Four times daily regimen have enabled to adjust the dose of Bu from the third dose onwards during the 1st day of Bu (depending on access to a biomedical analysis laboratory), which is not feasible with once daily dosing. However, TDM-guided dose adjustment from the 2nd day of Busulfan infusion is feasible with once-daily dosing and allows cumulative exposure to be readily estimated (75). The less commonly used twice daily Bu schedule (every 12 h administration, eight doses) allows dose adjustments from the 2nd day of Bu treatment, whilst reducing the workload associated with the four times daily dosing.

### Getting the First Dose of Busulfan Right: First Dose Personalization

When the use of TDM accounts for the interindividual PK variability of Busulfan, so allowing you to target the desired cumulative AUC, why is it important to individualise the first dose? Relying solely on TDM for dose adjustment has some limitations as well as having time-constrained limits on how quickly and how often dose adjustments can be made. Studies have highlighted the per-dose therapeutic window of Busulfan and the necessity to target early in administration the desired therapeutic window (39, 50, 66, 76). The personalization of the first dose of Busulfan should minimise the risk of overexposure and any associated acute toxicity. In combination with efficient TDM, this strategy could enable control of cumulative Busulfan exposure throughout conditioning treatment, which may optimise the outcomes. Because engraftment is associated with cumulative underexposure to Busulfan (50), first dose under-exposure seems to be less critical as it could be accounted for *via* TDM-guided dose adjustment. Even so, first dose underexposure could lead to the need for substantial dose augmentation, thus reaching a toxic C_max_ associated with SOS occurrence (50). This is particularly of concern in the case of once-daily dosing, where plasma concentrations reached are high and dose modifications are more considerable to correct the desired exposure in only four administered doses. Dividing the first dose into two half doses counteracts this risk and has been used successfully for many years in some centres (77).

The two strategies that can be implemented to personalise the first dose of Busulfan are the “test dose strategy” and the “first dose strategy.” The test dose strategy consists of the administration of a small dose of Busulfan ≥2 days before the start of the typical 4-day Busulfan conditioning course. This is particularly useful when the laboratory performing the Busulfan PK analysis is not on-site. The Busulfan PK obtained from the test dose is used to modify the first full dose according to the predicted PK and the chosen target exposure (78, 79). The first dose strategy consists of the personalization of the first dose according to the demographic and clinical attributes of the patient (age, weight, etc.). This strategy is based on dosing nomograms or algorithms derived from population PK studies. The advantage of this strategy is that it better considers each patient's individual characteristics for the recommendation of accurate first doses. As shown in Table 2, body size metrics (actual body weight, body surface area, fat-free mass, etc.) are covariates consistently reported to explain Busulfan PK variability in paediatric patients and are used for dose calculations (38, 57, 63, 66, 67, 80, 82–100).

**Table 2:** Summary of population PK models of busulfan.

**References**	***N* malignancy /*N* total**	**Age range (years)**	**Busulfan dosing**	**Structural model**	**Tested covariates**	**Included covariates**	**Final CL equation**	**Target daily exposure (AUC in mg.h/L)**	**Recommended initial dose**
**Model-informed dosing studies based on population PK models**									
Bartelink et al. (80, 81)	Model development: 114/245 Model validation: 39/158	0.1–35	IV q6 h, q12 h and q24 h	2 compartment model Linear elimination parameters	ABW, BSA, age, Supportive care treatments, baseline biological variables, diagnosis (malignancy vs. non-malignancy), dosing day	ABW for CL and V_d_ Dosing day for CL	CL_i_ = 3.32 (L/h) × (BW/ 15.3 kg)^1.57×BW^(−0.224)^^ × F_day2−4_	Target AUC: 22.5 Target window: 19.5–25.3	Bodyweight-based nomogram (80)
Ben Hassine et al. (82)	Model development: 191/302 Model validation: 67/100	0.1–20.1	IV q6 h, q12 h and q24 h	2 compartment model Linear elimination	ABW, age, sex, diagnosis (malignant vs. non-malignant), Fludarabine co-administration, the day of conditioning, *GSTA1* haplotypes, *GSTA1* metabolic capacity (three groups based on promoter haplotypes), Transplantation centre, treatment number.	ABW, PMA, the 1st day of conditioning, Fludarabine co-administration, and *GSTA1* metabolic capacity for CL. ABW for V_d_	CL_i_ = 4.92(*L*/*h*) × (*BW*/20*kg*)^1.14×PMA^(−0.20)^^ × *F*_day1_ × *F*_GSTA1_ × *F*_Fludarabine_	Target AUC: 19.7 Target window: 14.8–24.6	Dose (mg) = *AUC*_target_ × 4.92 (L/h) × (*BW*/20*kg*)^1.14×PMA^(−0.20)^^ × *F*_day1_ × *F*_GSTA1_ × *F*_Fludarabine_
Booth et al. (63)	15/24	0.3–16.7	IV q6 h	1 compartment, linear elimination	ABW, BSA, age	ABW for CL and V_d_	CL_i_ = 4.04 (L/h) × (ABW/20)^0.742^	Target AUC: 18.5 Target window: 14.8–22.2	For q6 h: ≤ 12 kg: 1.1 mg/kg/dose >12 kg: 0.8 mg/kg/dose
Choi et al. (83)	33/36	18–64	IV q6 h	1 compartment model with linear elimination	ABW, BSA, sex, drug interaction with azoles, AST, ALT, *GSTA1, GSTM1, GSTT1, GSTP1*	ABW and GSTA1(*A/*A vs. *A/*B) for CL	CL_i_ = 11.0 (L/h) × (BW/60 kg)^0.843^ × F*_*GSTA*1_*	Target AUC: NA Target window: 15.6–24.6	NA
Diestelhorst et al. (84)	Model Building: NR/82 Model Validation: NR/24	0.1–18.9	Model building: IV q6 h Model validation: IV q24 h	1 compartment model with linear elimination	ABW, BSA, age, height, sex	ABW for CL BSA for V_d_	CL_i_ = 3.04 (L/h) × (BW/16.1 kg)^0.797^	Target AUC: 18.8 Target window: NS	Dose (mg) = AUC_target_ × 3.04 (L/h) × (BW/16.1kg)^0.797^
Kawazoe et al. (85)	NR/54	0.3–53.5	IV q6 h	2 compartment model with linear elimination	Based on the model from McCune et al. (86)		CL_i_ = 11.8 (L/h) × (NFM_cl_/70 kg)^0.75^ × F_mat_ × F_T_CL_	Target AUC: NR Target window: 14.8–24.6	Dose (mg) = 11.8 (L/h) × (NFM_cl_/70kg)^0.75^ × F_mat_ × F_T_CL_
Langenhorst et al. (87)	231/385	0.16–73	IV	2 compartment model with linear elimination	ABW, BSA, age, supportive care treatments, baseline biological variables, diagnosis (malignancy vs. non-malignancy), dosing day	ABW for CL and V_d_ Dosing day for CL	CL_i_ = 7.48 (L/h) × (BW/43 kg)^1.03×BW^(−0.138)^^ × F_day2−4_	Target AUC: 22.5 Target window: 20.3–24.8	NA, only tested for TDM-guided cumulative exposure
Langenhorst et al. (87)	231/385	0.1673	IV	2 compartment model with linear elimination and a theoretical compartment for theoretical glutathione depletion	Based on Bartelink et al. (80, 81)	Based on Bartelink et al. (80, 81) + Age for GSH depletion factor.	CL_i_ = 7.61 (L/h) × (BW/43 kg)^1.04×BW^(−0.14)^^	Target AUC: 22.5 Target window: 20.3–24.8	NA, only tested for TDM-guided cumulative exposure
Long-Boyle et al. (88)	Model development: NR/90 Model validation: NR/21	0.124	IV q6 h	1 compartment model with non-linear elimination	ABW, BSA, height, age, sex, baseline biological variables	ABW for CL and V_d_ Age-dependent maturation for CL	<12 kg: CL_i_ = 4.32 (L/h) × (BW/22 kg)0.75 × (1+ Sl < bp × age) ≥12 kg: CL_i_ = 4.32 (L/h) × (BW/22 kg)0.75 × (1+ Sl<bp × Bp) × [1- Sl>bp × (age-12)]	Target AUC: 18.0 Target window: 14.4–21.6	<12 kg: Dose (mg) = AUCtarget ×4.32 (L/h) × (BW/22 kg)0.75 × (1+ 0.032 × age) ≥12 kg: Dose (mg) = AUCtarget ×4.32 (L/h) × (BW/22 kg)0.75 × (1+ 0.032 ×12) × [1+0.0138 × (age-12)]
McCune et al. (86)	978/1,481	0.1–65.8	IV q6 h, q8 h, q12 h, and q24 h	2 compartment model with linear elimination	ABW, height, post-menstrual age, age, sex, diagnosis (malignancy vs. non-malignancy), time since Bu treatment initiation	NFM (dependent of ABW, height and sex) for CL and V_d_ PMA-dependent maturation (Fmat) for CL Sex for V_d_ Time since Bu treatment initiation (FT_CL)	CL_i_ = 12.4 (L/h) × (NFM_cl_/70 kg)^0.75^ × F_mat_ × F_T_CL_	Target AUC: 18.5 Target window: 14.2–23.1	Dose (mg) = AUC_target_ ×12.4 (L/h) × (NFM_cl_/70 kg)^0.75^ × F_mat_
Nava et al. (89)	52/112	0.1–20	IV q6 h and q24 h	1 compartment, linear elimination	ABW, age, sex, diagnosis (malignant vs. non-malignant), co-administered chemotherapy, GSTA1 metabolic capacity (three groups based on promoter haplotypes)	ABW and PMA-dependent maturation (Fmat) for CL *GSTA1* metabolic capacity for CL PMA for V_d_	CL_i_ = 13.7 (L/h) × (BW/70 kg)^0.75^ × F_mat_ × F_GSTA1_	Target AUC: 18.5 Target window: 14.8–24.6	Dose (mg) = AUC_target_ ×13.7 (L/h) × (BW/70 kg)^0.75^ × F_mat_ × F_GSTA1_
Neely et al. (90)	Model building: NR/53 Model validation: NR/136	0.1–21	IV q6 h	1 compartment non-parametric model with linear elimination (estimated parameters are K_e_ and V_d_)	ABW, IBW, age	IBW and age for K_e_ and V_d_	CL = K_e_/V_d_ K_e_ = KeS×IBW−0.25 × (0.51 + 0.10×Age - 0.01 × Age2 + 0.00029 × Age3) V_d_ = VS × IBW × (0.71−0.016 × Age + 0.0017 × Age2)	Target AUC: 18.0 Target window: 14.4–21.6	For q6 h: ≤ 12 kg: 1.1 mg/kg >12 kg: 1.0 mg/kg
Nguyen et al. (57)	15/24	0.45–16.7	IV q6 h	1 compartment model with linear elimination	Height, age, BSA, ABW	ABW for CL and V_d_	CL_i_ = 2.97 (L/h) + 4.57 × [LN(ABW-3)]	Target AUC: 18.5 Target window: 14.8–24.6	For q6 h: <9 kg: 1.0 mg/kg/dose ≥9 to <16 kg: 1.2 mg/kg/dose ≥16 to <23 kg: 1.1 mg/kg/dose ≥23 to <34 kg: 0.95 mg/kg/dose ≥34 kg: 0.8 mg/kg/dose
Paci et al. (66)	82/115	0.1–15	IV q6 h	1 compartment model with linear elimination	ABW, BSA, age, sex, seizure prophylaxis, baseline biological variables	ABW for CL and V_d_	<9 kg: CLi = 2.18 (L/h) × (BW/9 Kg)1.26 >9 kg: CLi = ×2.18 (L/h) × (BW/9 Kg)0.76	Target AUC: 19.7 Target window: 14.8–24.6	<9 kg: Dose (mg) = AUCtarget ×2.18 (L/h) × (BW/9 Kg)1.26 >9 kg: Dose (mg) = AUCtarget ×2.18 (L/h) × (BW/9 Kg)0.76
Philippe et al. (91)	84/163	0.17–21	IV q6 h	1 compartment non-parametric model with linear elimination (estimated parameters are K_e_ and V_d_)	NA	IBW and age for K_e_ and V_d_	CL = Ke/Vd Ke = KeS × IBW−0.25 × (0.51 + 0.10 × Age - 0.01 × Age2 + 0.00029 × Age3) Vd = VS × IBW × (0.71−0.016 × Age + 0.0017 × Age2)	Target AUC: NA Target window: 14.8-24.6	Based on the highest cumulative probability of target interval attainment
Poinsignon et al. (92)	140/540 (75% model development and 25% model validation)	0.02–24.1	IV q6 h	1 compartment model with linear elimination	ABW, age	ABW and PMA-dependent maturation (F_mat_) for CL and V_d_	CL_i_ = 2.90 (L/h) × (BW/12 kg)^1.19×BW^(−0.134)^^ × F_mat_	Target AUC: 19.7 Target window: 14.8–24.6	For q6h: ≤ 11 kg: 1.15 mg/kg/dose >11 to ≤ 17 kg: 1.25 mg/kg/dose >17 to ≤ 25 kg: 1.05 mg/kg/dose >25 to ≤ 40 kg: 0.9 mg/kg/dose >40 kg: 0.8 mg/kg/dose
Rhee et al. (93)	NR/137 (70.8 % acute leukaemia)	0.6–22.2	IV q24 h	1 compartment model with linear elimination	ABW, BSA, age, height, sex, dosing day, baseline biological variables	BSA for CL and V_d_	CL_i_ = 10.7 (L/h) × (BSA/1.73)^1.07^ × (1-e^(−0.693/0.326)×*Age*^) × F_day_ × F_AST_	Target AUC: 18.75 Target window: 15.0–22.5	Age and BSA based nomogram [Rhee et al. (93)]
Savic et al. (67)	NR/149	0.1–3.3	IV q6 h and q24 h	1 compartment model with linear elimination	ABW, BSA, age, height, sex	ABW for CL and V_d_ Age-dependent maturation for CL	CL_i_ = 2.3 (L/h) × (Mat_mag_ + (1 – Mat_mag_) × [1 – *e* ^(−age × *Kmat*)^] × (BW/8 kg)^0.75^	Target AUC: 18.0 Target window: 14.4–21.6	Dose (mg) = AUC_target_ × (0.46 + (1 – 0.46) × [1 – *e* ^(−age×1.4)^] × (BW/8 kg)^0.75^
Shukla et al. (94)	Model building: NR/299 Model validation: NR/59	Model building: NR Model validation:0.2–20	IV q6 h, q12 h, and q24 h	1 compartment model with linear elimination	ABW, age, height, sex, dosing day, CloFluBu regimens	FFM based on ABW, height and sex for CL and V_d_ Age-dependent maturation for CL Day of conditioning CloFluBu regimens	CL_i_ = 3.96 (L/h) × (Mat_mag_ + (1 – Mat_mag_) × [1 – *e* ^(−age×*Kmat*)^] × (FFM/12 kg)^0.75^ × F_day1_ × F_regimen_	NA	Dose (mg) = AUC_target_ ×3.96 (L/h) × (Mat_mag_ + (1 – Mat_mag_) × [1 – *e* ^(−age×*Kmat*)^] × (FFM/12 kg)^0.75^ × F_day1_ × F_regimen_
Trame et al. (95) BSA based	NR/94	0.1–18.8	Oral q6 h IV q24 h	1 compartment model with linear elimination	ABW, BSA, age	BSA for CL	CL_i_ = 4.16 (L/h) × BSA	Target AUC: 18.8 Target window: 14.8–24.6	Dose (mg) = AUC_target_ ×4.16 (L/h) × BSA
Trame et al. (95) weight based	NR/94	0.1–18.8	Oral q6 h IV q24 h	1 compartment model with linear elimination	ABW, BSA, age	ABW for CL	CL_i_ = 4.11 (L/h) × (ABW/27.2)^0.75^	Target AUC: 18.8 Target window: 14.8–24.6	Dose (mg) = AUC_target_ ×4.11 (L/h) × (BW/27.2 kg)^0.75^
Wu et al. (96)	53/53	7.0–59.0	IV q6 h	1 compartment model with linear elimination	ABW, BMI, AIBW, BSA, sex, serum creatinine	BSA for CL and Vd	CL = 11.1 (L/h) × (BSA/1.587)^0.955^	NA	Dose (mg) = AUC_target_ ×11.1 (L/h) × (BSA/1.587)^0.955^
Yuan et al. (97)	Model building: 26/69 Model validation: 4/14	0.5–15.2	IV q6 h	1 compartment model with linear elimination	BSA, AST, GSTA1 (*A/*A vs. *A/*B)	BSA for CL and V_d_ AST and *GSTA1* for CL	CL = 4.92 (L/h) × (BSA/0.67)^0.83^ × (AST/29.10)^−0.21^ × F*_*GSTA*1_*	Target AUC: 18.5 Target window: 14.8–22.2	GSTA1-*A/*A: BSA 0.2–0.4 m^2^: 45 mg/m^2^ BSA 0.4–0.7 m^2^: 42 mg/m^2^ BSA 0.7–1.6 m^2^: 38 mg/m^2^ GSTA1-*A/*B: BSA 0.2–0.4 m^2^: 40 mg/m^2^ BSA 0.4–0.7 m^2^: 37 mg/m^2^ BSA 0.7–1.6 m^2^: 34 mg/m^2^
Zwaveling et al. (98)	35/77	0.2–23	IV q6 h and q24 h	1 compartment model with linear elimination	ABW, BSA, Age, diagnosis (malignant vs. non-malignant) *GSTA1*, *GSTM1*, *GSTP1*, *GSTT1*	ABW for CL and V_d_	CL_i_ = 4.8 (L/h) × (ABW/19)^0.84^	NA	NA
**Dosing recommendations not based on population PK studies**									
Ansari et al. (38)	75	0.1–20	IV q6 h	NA	NA	NA	NA	Target window: 14.4–21.6	For q6h: <3 months: 16 mg/m2/dose >3 months to <1 year: 0.8 mg/kg/dose >1 year old to <4 years old: 1 mg/kg/dose >4 years old: 0.8 mg/kg/dose
Buffery et al. (99)	150	0.5–58	Oral or IV q6 h IV q24 h	NA	NA	NA	NA	Target window: 15.2–22.2 in children, 14.8–23.0 in adults	For q6h: 10–16 kg: 1.2 mg/kg/dose 17–18 kg: 1.1 mg/kg/dose 19–22 kg: 1 mg/kg/dose 23–25 kg: 0.9 mg/kg/dose >26 kg: 0.8 mg/kg/dose
Wall et al. (100)	24	0.5–16.7	IV q6 h	NA	NA	NA	NA	Target window: 14.8–22.2	For q6h: <4 years: 1 mg/kg/dose ≥4 years: 0.8 mg/kg/dose

*ABW, actual body weight; AIBW, adjusted ideal body weight; ALT, alanine aminotransferase; AST, aspartate aminotransferase; AUC, area under the curve; BMI, body mass index; BSA, body surface area; Bu, busulfan; BW, body weight; CL, clearance; C_max_, maximum concentration; F, fraction absorbed (bioavailability); FFM, fat-free mass; GSTA1, glutathione S-transferase A1; i, intrinsic; IBW, ideal body weight; IV, intravenous; K_e_, elimination rate constant; mag, magnitude; mat, maturation; LN, natural logarithm; NA: Not applicable; NFM, normal fat mass; NR, not reported; PMA, post-menstrual age; q12h, every 12 hours; q24h, every 24 hours; q6h, every 6 hours; q8h, every 8 hours; V_d_, volume of distribution*.

Several studies have also included an age-based metric to describe the ontogeny and maturation of Busulfan clearance. Such a model has been shown to result in accurate PK predictions and selection of the first dose in paediatric patients (75, 101–103).

For both the test dose and first dose strategies, intraindividual (i.e., inter-day) PK variability of Busulfan mandates that repeat PK testing is needed to assess the cumulative AUC over the course of therapy (78–80, 82, 104). In this way, personalised first doses coupled with efficient TDM permits the desired Busulfan exposure to be targeted. More importantly, repeat measurements used for TDM allow the cumulative exposure to be measured: this can inform future studies, particularly as additional drugs are added to the backbone of a Busulfan-based conditioning, so optimising the outcome and minimising the risk of toxicities related to under- or over-exposure.

### The Role of Pharmacogenomics in the Pharmacokinetics and Pharmacodynamics of Busulfan-Based Chemoconditioning

In recent years, in an effort to accurately predict Busulfan PK in paediatric patients, the influence of biomarkers explaining Busulfan PK became an area of interest. Table 3 summarises the studies on the association between pharmacogenetic markers and Busulfan PK in paediatric HSCT patients (39, 71, 82, 83, 89, 97, 98, 102, 105–107, 109–120).

**Table 3:** Summary of studies assessing busulfan pharmacogenetics and pharmacokinetics.

**References**	**N ALL/N total**	**Age range (years)**	**Conditioning regimen(s)**	**Tested marker(s)**	**Tested Bu PK parameters in relation to the marker**	**PK findings**	**Clinical findings in relation to the biomarker**
Abbasi et al. (105)	0/185 (48 AML patients)	0.5–66	IV Bu (*N* = 57): q12 h or q6 h Oral Bu (*N* = 128): q6 h Combinations with Cy, Flu, Thio, VP16, Mel	*GSTA1* *GSTM1*	CL Dose adjustments	No association with IV Bu Decreased CL of oral Bu in GSTA1*B individuals	NA
Ansari et al. (106)	2/28	0.4–19.8	q6 h IV Bu with Cy	*GSTA1* *GSTP1* *GSTM1*	AUC C_max_ Css CL	*GSTM1-null* genotype associated with: 1.2-fold higher AUC 1.3-fold higher C_max_ 1.2-fold higher Css 1.3-fold lower CL	NA
Ansari et al. (107)	6/69	0.1–19.9	q6 h IV Bu: BuCy BuCyVP16 BuMel	*GSTA1 GSTP1 GSTM1*	C_max_ AUC Css CL	Higher CL in presence of GSTA1-*A2 Lower CL with *GSTM1-null* in patients >4 years	Higher risk of SOS with *GSTA1* homozygous and heterozygous *B1b (HR 10 and 5.6, respectively) 4-fold higher risk of aGVHD with *GSTM1-null* in patients >4 years
Ansari et al. (108)	0/44 (only thalassaemic patients)	1.5–17	q6 h IV Bu with Cy	*GSTA1 GSTM1*	Css Cmax AUC CL	Higher CL in presence of GSTA1-*A Higher Bu exposure and lower clearance in GSTA1-*B/*B patients (*p* ≤ 0.01)	5-fold higher risk of aGVHD and TRT with *GSTM1-null*
Ansari et al. (39)	12/138	0.1–9.9	q6 h IV Bu with other agents (Cy, Mel, VP16)	*GSTA1* *GSTM1* *GSTP1*	C_max_ C^ss^ AUC_cum_ CL Initial/adjusted dose ratio	Higher CL and lower AUC_cum_ with *GSTA1* diplotypes associated with rapid metabolising capacity Lower CL and higher AUC_cum_ with *GSTA1* diplotypes associated with slow metabolising capacity Lower CL in patients >4 years with *GSTM1-null*	Higher incidence of SOS, aGvHD and combined TRT, with *GSTA1* diplotypes with slow metabolising capacity *GSTP1 313GG* associated with acute GvHD grade I–IV *GSTM1-non-null* genotype associated with HC
Ben Hassine et al. (82)	44/402 (302 for model building, 100 for model validation)	0.1– 20.1	q24 h, q12 h, q6 h IV Bu with other agents	*GSTA1*	CL V_d_	*GSTA1-G3* (slow metabolising capacity) associated with 12% lower CL *GSTA1-G1* (rapid metabolising capacity) associated with 10% higher CL	NA
Bonifazi et al. (109)	35/185 patients received Bu	18–59	q6 h IV Bu with Cy or Flu	30 genes including *GSTA1* *GSTM1* *GSTT1* *GSTA2*	AUC	1.5-fold higher AUC in *GSTA2 S112T* serine/serine patients compared to threonine amino acid substitution patients	NA
Bremer et al. (110)	13/114	16–65	q6h IV Bu with Cy	*GSTA1 GSTT1 GSTM1 GSTP1*	CL/F Css	CL/F 11% and 18% lower when 1 or 2 GSTA1-*B alleles are present, respectively. 60% higher Css with GSTA1-*B/*B and *GSTT1/GSTM1 double-null*	Higher mortality within the first 30 days post-HSCT with *GSTM1-null*
Choi et al. (83)	13/36	18–64	q6 h or q24 h IV Bu with Cy or Flu	*GSTA1 GSTT1 GSTM1 GSTP1*	CL AUC	15% lower CL in heterozygous GSTA1-*B	NA
Elhasid et al. (111)	0/18 (only congenital haemoglobinopathies)	0.8–16	Oral Bu q6h	*GSTA1 GSTT1 GSTM1 GSTP1*	Cmax AUC AUC/kg CL/F T_1/2_ Vd/F Cmax/AUC ratio	Association between *GSTA1* and *GSTP1* genotypes with C_max_ and AUC	Association between *GSTM1-null* genotype with acute/chronic GvHD and with graft rejection
Gaziev et al. (112)	0/71 (only thalassaemic patients)	1.6–27	q6 h IV Bu with Cy or Thio	*GSTA1 GSTT1 GSTM1 GSTP1*	Css AUC CL T_1/2_	10% lower CL in patients carrying GSTA1*B	NA
Johnson et al. (113)	2/29	0.1–18.3	q6 h or q12 h IV Bu with Cy or Flu	*GSTA1 GSTM1 GSTP1*	CL AUC Css C_max_	30% lower CL with GSTA1-*B or *B/*B Significant differences in AUC, Css and C_max_ between GSTA1-*A/*A, *A/*B and *B/*B genotypes (lower exposures with *A/*A and higher exposures with *B/*B)	NA
Kim et al. (114)	6/58	16–58	q6 h IV Bu alone or with Cy or Flu	*GSTA1 GSTT1 GSTM1*	CL AUC	Higher AUCs with GSTA1-*A Lower Bu CL in *GSTM1/GSTT1-double-null* patients	NA
Lee et al. (71)	7/24	0.9–18.1	q24 h IV Bu with Flu. VP16 was added for ALL patients	*GSTA1 GSTT1 GSTM1*	AUC first-day CL Dose modification	NS Tendency of higher AUC in carriers of GSTA1-*A/*B genotype or *GSTT1-null* genotype	NA
Nava et al. (102)	10/101	0.1–21.0	q6 h IV Bu-based conditioning: BuCy BuFlu BuCyVP16 BuMel	*GSTA1*	CL AUC	*GSTA1*-diplotype-based metabolic groups associated with the mean prediction error of CL CyGSTA1 slow metabolising capacity associated with AUCs within therapeutic window GSTA1 rapid metabolising capacity associated with subtherapeutic AUCs	NA
Nava et al. (89)	8/112	0.1–20.0	q6 h and q24 h IV Bu-based conditioning: BuCy BuCyVP16 BuMel BuCyMel BuMelAraC	*GSTA1*	CL V_d_ AUC first-dose	*GSTA1*-G3 (slow metabolising capacity) associated with 11% lower CL *GSTA1*-G1 (rapid metabolising capacity) associated with 7% higher CL Doses considering *GSTA1* resulted in no G1 patients outside the target AUC	NA
Nishikawa et al. (115)	0/20 (9 AML patients)	0.5–17	q6 h IV Bu with other agents (Cy, Flu, Mel, VP16)	*GSTA1 GSTT1 GSTM1*	CL AUC K_e_	Poor metabolizers, defined as patients carrying ≥1 GSTA1-*B or *GSTM1-double-null* genotypes, had lower 28%, lower CL and 52% higher AUC than extensive metabolizers	NA
Srivastava et al. (116)	0/114 (only thalassaemic patients)	2–16	q6 h oral Bu with Cy	*GSTM1 GSTT1*	CL/F Css	Lower Bu CL/F with *GSTM1-null*	3-fold higher risk of SOS with *GSTM1-null*
ten Brink et al. (117)	NR/84 (31 patients with haematological malignancies including ALL)	Mean 6.1 years (± 5.4 SD)	q24 h IV Bu with Cy or Flu and other agents (Cy or Flu, Thio, Mel, VP16, Clo)	*GSTA1* *ABCB4* *CYP39A1* *CYP2C19* *SLC7A8* *SLC22A4*	CL AUC	8% lower CL with GSTA1-*A/*B and 26% lower CL with GSTA1-*B/*B compared to wild-type (*A/*A), with a larger effect of *GSTA1* in patients <2 years of age 13% lower CL With heterozygous *CYP39A1* variant and 17% lower clearance with homozygous mutant *CYP39A1* 39% lower CL with homozygous carriers for both haplotypes of *GSTA1* and *CYP39A1*	NA
Uppugunduri et al. (118)	6/66	0.1–19.9	q6 h IV Bu-based conditioning: BuCy BuFlu BuCyVP16 BuMel	*CYP2C9* *CYP2C19* *CYP2B6* *FMO3*	Bu/sulfolane metabolic ratio	Higher metabolic ratio in CYP2C9*2 and *3 (decreased function) allele carriers Lower metabolic ratio in CYP2C19*17 (increased function) allele carriers	Higher metabolic ratio (<5) associated with lower graft failure risk Higher incidence of relapse and graft failure in patients with malignant disease with homozygous reduced-function *CYP2B6* alleles
Yin et al. (119)	8/25	13–61	q6 h IV Bu with other agents (Cy, Flu, Mel, VP16, AraC, Decitabine, Semustine)	*GSTA1 GSTP1*	AUC CL C_max_ T_1/2_ V_d_	Lower CL and higher exposure in GSTA1-*A/*B patients compared with *A/*A patients Higher CL in presence of GSTP1 313A-*G (dominant allele)	NS
Yuan et al. (97)	5/69 (model building) + R/14 (model validation)	0.5–15.8	q6 h IV Bu with other agents (Cy, Flu, Mel, VP16, AraC, decitabine, semustine)	*GSTA1*	CL AUC_0−6*h*_	17% lower CL in heterozygous GSTA1-*B	Worse neutrophil recovery and lower survival in heterozygous GSTA1-*B patients
Zwaveling et al. (98)	NR/77 (35 patients with malignancies)	0.2–23	q24 h or q6 h IV Bu with other agents (Cy, Mel, Flu, VP16)	*GSTA1 GSTT1 GSTM1 GSTP1*	CL	NS	1.7-fold higher risk of SOS in *GSTM1-null* patients (trend, *p* = 0.07)

*ALL, acute lymphoblastic leukaemia; AML, acute lymphoblastic leukaemia; AUC, area under the curve; AUC_cum_, cumulative area under the curve; Bu, busulfan; CL, clearance; C_max_, maximum concentration; Css, steady state concentration; Cy, cyclophosphamide; F, fraction absorbed (bioavailability); Flu, fludarabine; GSTA1, glutathione S-transferase A1; GvHD, graft-versus-host disease; HC, haemorrhagic cystitis; HR, hazard ratio; IV, intravenous; K_e_, elimination rate constant; Mel, melphalan; NS, not significant; NR, not reported; q12h, every 12 hours; q24h, every 24 hours; q6h, every 6 hours; q8h, every 8 hours; SOS, sinusoidal obstruction syndrome; T_1/2_, half-life; Thio, thiotepa; TRT, treatment-related toxicity; V_d_, volume of distribution; VP16, etoposide*.

As Busulfan is mainly metabolised by glutathione-S-transferases (GSTs) (121, 122), clinical investigations on the influence of genetic polymorphisms related to GST activity on Busulfan PK were initiated in the early 2000's (116). Table 3 shows that Busulfan PK is mainly associated with haplotypes of the promoter regions of *GSTA1* (18 studies) and *GSTM1* (7 studies). The association between *GSTP1* and *GSTT1* with Busulfan PK is scarce, probably due to their less important role in Busulfan metabolism compared with A1 and M1 isoforms (123). GSTA1-^*^B haplotypes have been associated with decreased Busulfan clearance, implying an increased exposure to Busulfan. This is due to decreased *GSTA1* expression with ^*^B haplotypes (39, 124). Initially, ^*^A and ^*^B haplotypes of GSTs were determined using one single nucleotide polymorphism (SNP) (either *52G/A rs3957356* or -*69C/T rs3957357*, in linkage disequilibrium) (113, 116, 117). The association of these haplotypes with Busulfan PK are still being studied (97). More recently, *GSTA1* haplotypes have been shown to be more complex, requiring the genotyping of at least four SNPs of the *GSTA1* promoter (39, 82, 124). In fact, sub-haplotypes within ^*^A and ^*^B have significantly different gene expression potentials. Within ^*^A haplotypes, the ^*^A1 sub-haplotype has a decreased expression potential than ^*^A2 and ^*^A3 haplotypes. The ^*^A2 haplotype has been associated with a significantly increased clearance and thus lower Busulfan exposure (108). Within ^*^B haplotypes, which are all associated with poor Busulfan metabolism, patients carrying the sub-haplotype ^*^B1b have significantly decreased Busulfan metabolism and clearance compared with other ^*^B haplotypes (39).

These different gene expression potentials have enabled the classification of patients into three (82, 89, 102, 124) or four (39) groups according to their capacity to metabolise Busulfan. *GSTA1* polymorphisms have been also associated with the clinical outcome of HSCT (SOS, acute GvHD, transplant-related mortality, engraftment, and survival) (39, 97, 107, 108). These associations are likely to be related to differing exposure to Busulfan according to the *GSTA1* haplotype. More recently, genetic polymorphisms explaining the metabolising capacity of GSTA1 have been detected as a significant covariate influencing Busulfan clearance: two recent models included as significant covariates GSTA1 metabolic groups associated with Busulfan metabolic capacity, based on *GSTA1* sub-haplotypes (82, 89). Predictions based on these models have enabled researchers to accurately achieve Busulfan AUC within the Busulfan EMA therapeutic window in around 80% of the patients from an independent cohort of which 13% of patients had ALL (82). The addition of GSTA1 metabolic capacity to the model seems to have improved the accuracy of first dose selection.

The pharmacogenomic-based models are likely to enable accurate targeting of Busulfan exposure from the beginning of Busulfan conditioning, limiting the need for dose adjustments. A prospective validation is still required for the implementation of this dosing recommendation, although the model was validated in an external cohort. In addition, the feasibility of the implementation of *GSTA1* genotyping in routine clinical practise needs to be assessed. These aspects are being addressed in the current BuGenes01 multicentre, prospective randomised trial (Clinicaltrials.gov identifier: NCT04822532), in which paediatric patients undergoing HSCT will be randomised to either a pharmacogenomic-based first dose algorithm or the best-performing dosing algorithm currently used (86). Personalising the first dose of Busulfan in paediatric patients should enable researchers to appraise the unpredictability of Busulfan PK, thus limiting large dose adjustments that could subsequently overexpose these patients (86).

*GST* polymorphisms have also been associated with poor HSCT outcomes and TRT, as shown in Table 3. These associations were reported in patients carrying *GST* haplotypes expressing poor metabolising phenotypes, for example GSTA1^*^B, GSTM1-null, and GSTP1 313^*^G haplotypes. Polymorphisms of *GSTA1, GSTM1*, and *GSTP1* were reported as risk factors for SOS (39, 107, 116, 125) and acute GvHD (39, 107, 108, 111), while *GSTA1* and *GSTM1* have been associated with combined TRTs (39, 107, 108). *GSTM1* was associated with graft rejection and mortality within 30 days post-transplant (111), while *GSTA1* was associated with neutrophil recovery and survival (97). Whether these associations are solely due to the influence of *GST* polymorphisms on Busulfan PK is questionable. In a study by Ansari et al., increased TRT was associated with *GSTA1* polymorphisms in multivariate logistic regression even when Busulfan exposure was accounted for (39). *GSTA1* seems to have a direct influence on the transplant outcomes in addition to influencing Busulfan PK. Furthermore, the same study demonstrated that, in patients within or below the therapeutic window (Css 600 – 900 ng/ml, corresponding to daily AUC of 14.4 −21.6 mg.h/L), *GSTA1* haplotypes expressing poor metabolic capacity were associated with higher TRT risk (HR 4.4; *p* < 0.0005) (39). This association was not observed in patients overexposed to Busulfan (Css >900 ng/mL) for whom TRT rates were very high irrespective of the *GSTA1* genotype. This suggests that when patients are within therapeutic exposures, the influence of the poor metabolising capacity of *GSTA1* on TRT occurrence is independent of PK. *GST* polymorphisms could therefore influence toxicities and outcomes of HSCT independently of Busulfan exposure. This aspect should be further explored in future studies of patients receiving Busulfan.

Other genetic markers for Busulfan conditioning toxicities have been reported. In paediatric patients, *CYP2B6, CTH, MTHFR, HPSE, UGT2B10*, and *KIAA1715* were reported as risk factors for SOS (126). The risk related to the combined presence of these markers remains to be studied further. Interestingly, *CTH c.1364 TT*, a gene coding for cystathionase (an enzyme that participates in the glutathione synthesis pathway), was reported to be associated with SOS risk in combination with GSTA1^*^B^*^B (reduced function) (127). The data from the pharmacogenomic add-on study of the FORUM study will address this question. Recent studies have reported that polymorphisms of *MGMT* (128)*, ERC1, PLEK, NOP9*, and *SPRED1* were associated with increased GvHD risk (129) in paediatric HSCT, both studies included ALL patients. Donor polymorphisms of genes encoding interleukins (ILs), such as IL-6, interferon γ (IFNγ), and IL-7Rα, have also been associated with GvHD in studies including adult and paediatric patients receiving HSCT, both studies including ALL diagnoses (130, 131). The inclusion of these genetic variants in prognostic models for TRTs could be useful to guide personalised interventions. Combined with other known risk factors for SOS, genetic markers for increased risk of SOS could aid the selection of reduced toxicity chemo-conditioning regimens (e.g., those composed of maximum of two alkylating agents, or/and Fludarabine based), and the administration of defibrotide prophylaxis. Furthermore, the presence of markers of increased GvHD risk could contribute to the choice of GvHD prophylaxis.

## Optimising the Use of Treosulfan

Unlike Busulfan, Treosulfan is a prodrug—to gain cytotoxic activity it has to undergo non-enzymatic pH and temperature dependent transformation to biologically active metabolites—which takes place spontaneously under physiological conditions, without involvement of hepatic metabolism. These epoxy derivates of Treosulfan mediate DNA alkylation and interstrand cross-linking (132, 133).

Due to its strong antineoplastic, myeloablative and immunosuppressive properties as well as favourable toxicity profile, the use of Treosulfan in paediatric HSCT conditioning has grown rapidly. In 2019 it was authorised by the EMA for use as a conditioning treatment in adults and children from 1 month of age.

Much of the early literature on Treosulfan-based conditioning comes from its use in non-malignant disease. High rates of engraftment and low non-regimen-related toxicity have translated into good survival rates (134–136). Commonly encountered regimen-related toxicities include skin toxicity and mild mucosal toxicity (137, 138). Importantly for use in malignant disease, there is a low rate of VOD (137–139); specifically, there is a much lower rate compared with Busulfan in high-risk beta thalassaemia patients (30 vs. 78%, respectively) (140).

An additional and major potential long-term benefit of Treosulfan-based conditioning is that it may be less gonadotoxic than Busulfan (141). Higher rates of spontaneous puberty and menarche and lower luteinizing hormone levels in patients receiving Treosulfan vs. Busulfan all suggest less damage to the gonad; there is hope that this will translate to fertility and pregnancies in the future.

A summary of the use of Treosulfan in malignant disease can be found in Table 4 (34, 142–145).

**Table 4:** Summary of studies assessing the use of treosulfan conditioning in children with malignant diseases.

**References**	**ALL (N) / study population (N)**	**Age range (years)**	**Conditioning regimen(s)**	**Treo dose**	**Tested outcome(s)**	**Toxicity (grade ≥III)**
Wachowiak et al. (142); retrospective	17/51	0.7–17 (median 8)	TreoVP16Cy (25%) TreoFluMel (18%) TreoCyMel (16%) TreoCy (18%) TreoFlu (18%) TreoMel (6%)	30–42 g/m^2^	Engraftment: 94% Graft failure: 6% CC: 90% RI: 22% DFS: myeloid malignancy: 71% lymphoid malignancies: 41%	Day +100: Mucosal: 12% Renal: 2%
Beier et al. (143); retrospective	16/109	0–18	TreoFluThio (43%) TreoFlu (31%) TreoFluMel (15%) TreoMel (4%) TreoCy (2%) TreoMelCy (2%) TreoFluCy (1%)	21–42 g/m^2^	Engraftment: 100% OS in malignant group: 49% TRM: 11.9%	Skin grade IV: 3.5% Pulmonary grade IV: 2%
Boztug et al. (144); retrospective	71/193	0.4–18 (median 9.1)	TreoFluThio 33% TreoCy 25% TreoFlu 22% TreoFluMel 13% Other 7%	33–45 g/m^2^	^*^3-year OS: 51% ^*^3-year EFS: 39% ^*^TRM: 14%	^*^Stomatitis: 36% ^*^Diarrhoea: 24% ^*^Vomiting: 11% ^*^Respiratory toxicity: 14% ^*^Elevated bilirubin: 14% ^*^Elevated SGOT: 27% ^*^CNS toxicity: 4% ^*^Peripheral neurotoxicity: 4% ^*^VOD: 0%
Kalwak et al. (145); prospective, Phase II	23/65	1–17 (median 12)	TreoFluThio	30–42 g/m^2^	Engraftment: 98.5% CC at Day +100: 92.2% ^*^OS: 78.3% ^*^RI: 26.1% ^*^R/PFS: 69.6% NRM: 3.1%	Mucositis oral: 43.1% Nausea and vomiting: 16.9% Infections and infestations: 30.8% Diarrhoea: 15.4% Skin and subcutaneous: 12.3% VOD: 0%
Peters et al. (34); prospective, Phase III	93/93	^*^4–18	^*^TreoFluThio	^*^42 g/m^2^	^*^OS: 77% ^*^EFS: 58% ^*^CIR: 31% ^*^TRM: 12%	^*^Vomiting: 20% ^*^Stomatitis: 56% ^*^Infection: 65% ^*^Peripheral neurotoxicity: 6% ^*^HLH: 3% ^*^PTLD 7% ^*^Skin changes: 9% ^*^Aspiration: 4%

* *Data specific to the subgroup of patients with ALL*.

*ALL, acute lymphoblastic leukaemia; Bu, busulfan; CC, complete donor chimerism; DFS, disease-free survival; EFS, event-free survival; Flu, fludarabine; HLH, haemophagocytic lymphohistiocytosis; Mel, melphalan; NRM, non-relapse mortality; OS, overall survival; PTLD, post-transplant lymphoproliferative disorder; R/PFS, relapse/progression-free survival; RI, relapse incidence; SGOT, serum glutamic oxaloacetic transaminase; SOS, sinusoidal obstruction syndrome; Thio, thiotepa; Treo, treosulfan; TRM, treatment-related mortality; TRT, treatment-related toxicity; VOD, veno-occlusive disease; VP16, etoposide*.

### Toxicity of Treosulfan-Based Conditioning

Prior to the FORUM study, published experience of Treosulfan use in patients with ALL was scarce. Wachowiak et al. retrospectively evaluated 51 children with high risk or advanced haematological malignancies (17 with ALL) transplanted between 2000 and 2005 with Treosulfan-containing conditioning regimens and found no early regimen-related fatal toxicity and a NRM of 16% at 4 years (142). In a retrospective analysis of 109 children transplanted using Treosulfan-based conditioning between 2003 and 2009, approximately half of children had malignancy and 16 had ALL. Treosulfan was combined with agents such as Fludarabine, Thiotepa, and Melphalan. Skin toxicity was frequent but mild with Treosulfan, mucosal toxicity was reduced compared with Busulfan, VOD occurred in 3%, and seizures in 4% of patients (143). Boztug et al.'s retrospective study of 193 children and adolescents with malignant haematological disorders who received HSCT after Treosulfan-based conditioning therapy included 71 with ALL. In accordance with previous studies, toxicity of Treosulfan was low and mainly gastrointestinal in this study. VOD and neurological toxicity were rare. No association of toxicity with type of disease or Treosulfan dose was found. TRM was at 14% (144).

In a Phase II, prospective, multicentre study conducted by Kalwak et al., Treosulfan-Fludarabine-Thiotepa conditioning was investigated in 65 children with a haematological malignancy (3 ALL, 29 AML, 10 myelodysplastic syndrome and 3 juvenile myelomonocytic leukaemia). Treosulfan was dosed by body surface area (BSA), with those patients ≤ 0.5 m^2^ receiving 10 g/m^2^/day; those >0.5–1.0 m^2^ 12 g/m^2^/day and those >1.0 m^2^ 14 g/m^2^/day for 3 days. Overall, 98.5% of patients achieved engraftment, with complete donor chimerism in 92.6% at 12 months. The most frequently reported toxicities of grade 3–4 were oral mucositis (43.1%), infections (30.8%), nausea and vomiting (16.9%), skin and subcutaneous tissue disorders (12.3%), and hepatic VOD (1.4%). NRM was estimated to be low, at 3.1% (145).

To date, only preliminary results of the Treosulfan arm in the FORUM trial have been published: the most frequent early grade 3–4 toxicities included infections (65%) and stomatitis (56%), while skin toxicity of grade 3–4 was present in 9% of patients. Of concern, neither the Treosulfan nor Busulfan arm compared favourably with TBI with regards to TRM in the modified as-treated population (12, 6, and 3%, respectively; *p* = 0.1103). Analysing the two chemotherapy groups together, the higher TRM compared to the TBI arm (9 vs. 2%, *p* = 0.027) contributed to the lower overall survival, triggering the cessation of randomisation to the chemotherapy arms (34). This raises concerns of duplicating what was seen in the PBMTC study (20), with a more intensive and thus toxic combination of chemotherapy agents not comparing favourably with the well-known early toxicity profile of TBI.

### Outcome Data for Treosulfan-Based Conditioning in Paediatric ALL HSCT

Prior to the FORUM trial, children with ALL receiving Treosulfan-based conditioning therapy prior to HSCT were reported in cohorts together with non-malignant disorders (143) or with other (myeloid) malignancies (142, 145). The numbers of paediatric ALL patients included in trials did not exceed 71 in retrospective cohorts (144) or 23 in prospective trials (145). The more robust outcome data for Treosulfan-based conditioning come from studies with myeloid malignancies in adults (146–148).

In the retrospective study of Wachowiak et al. referred to above, the estimated 4-year probability of DFS was 71% for those with myeloid malignancies and 41% in the 20 patients with lymphoid malignancies (predominately ALL), with an acceptable relapse incidence of 24% at 4 years (142). Beier et al., in a cohort including 16 patients with ALL and 11 with AML, reported a 3-year EFS of 49% and with predominant cause of death being relapse (143). In the European Society for Bone and Marrow Transplantation (EBMT) Paediatric Diseases Working Party retrospective analysis of Treosulfan-based conditioning for Haematological malignancy, the 3-year EFS was 45% and disease-related mortality 32% for the 71 ALL patients (144). The addition of an additional alkylator (either Thiotepa or Melphalan) to the Treosulfan-Fludarabine backbone resulted in significantly better OS.

One should bear in mind that these early retrospective studies selected patients who were felt to be at high risk for regimen-related toxicity, especially pulmonary and hepatic (VOD) toxicity associated with standard of care myeloablative regimes (TBI or Busulfan based). In 23 prospectively studied paediatric ALL patients given Treosulfan-Fludarabine-Thiotepa, Kalwak et al. estimated the relapse/progression incidence to be 26.1%, the relapse/progression free survival to be 69.9% and OS to be 78.3% at 36 months (145). Outcomes were comparable across each of the BSA-based Treosulfan doses (10, 12, and 14g/m^2^).

The most valuable knowledge on the efficacy of Treosulfan-Fludarabine-Thiotepa conditioning before HSCT for paediatric ALL comes from the 99 patients with ALL randomised to this regimen in the FORUM trial (34). Outcomes in the Treosulfan arm in the modified as-treated population-−58% EFS, 77% OS, 31% cumulative incidence of relapse and 12% TRM at 2 years—were significantly lower than the TBI arm (85% EFS, 91% OS, 12% cumulative incidence of relapse and 3% TRM at 2 years), clearly not supporting the use of an unadjusted Treosulfan regimen for patients eligible for TBI.

### Treosulfan Pharmacokinetics and Outcome

One difference between the Busulfan and Treosulfan arms in the FORUM study is that we know a significant proportion of patients in the Busulfan arm will have had PK analysis performed, with subsequent TDM. In contrast, we do not expect any of the Treosulfan-assigned patients to have had TDM. A fundamental question remains unanswered: is there a meaningful relationship between drug exposure and clinical outcome for Treosulfan and will optimization of dose and TDM improve the EFS vs. TBI when compared with the non-TDM-targeted Treosulfan usage in FORUM?

We know that, like most of the drugs we use in conditioning, there is high inter-patient variability in exposure to Treosulfan (149). To date, most of the PK data for Treosulfan was collected in patients with non-malignant disease. Van der Stoep et al. performed a prospective multicentre study in 77 children undergoing HSCT (84.4% of whom had non-malignant disease), focussing on the PK profile of Treosulfan. Their results showed that there is a relationship between Treosulfan exposure and early toxicity. Patients with higher exposure (AUC >1,650 mg.h/L) had an increased risk of developing grade 2 or higher mucositis and skin toxicity. No correlation between Treosulfan exposure and the early clinical outcome parameters (engraftment, acute GvHD or donor chimerism) was found (149). A prospective study in two UK centres looked at Treosulfan PK and PD in children undergoing allogeneic HSCT mainly for primary immunodeficiency after Treosulfan-Fludarabine conditioning. An association between high AUC and mortality as well as low AUC and poor engraftment was shown (150).

Mohanan et al. studied 87 patients with thalassaemia major undergoing allogeneic HSCT. Treosulfan clearance of <7.97 L/h/m^2^ was significantly associated with poor OS and EFS; where as high Treosulfan clearance (>7.97 L/h/m2) and low AUC (<1,828 mg.h/L) showed a trend toward better OS (151).

Thus, it can be postulated that there is likely to be an association of outcome and toxicity parameters with Treosulfan exposure, yet perhaps the improved safety profile of Treosulfan over Busulfan makes this more difficult to establish until we have available larger studies on more uniform populations. In most protocols, Treosulfan is administered over 3 consecutive days in doses of 10–14 g/m^2^/day, with the dose adjusted according to age or body weight. Despite the dose reduction to 10 g/m^2^ in infants, admittedly with a variety of diagnoses, including many with non-malignant disease, Treosulfan exposure remained higher compared with older children receiving 14 g/m^2^ (149). We may find that it is in these younger (and so smaller) patients where Treosulfan TDM has a role. In order to identify and quantify sources of variability in drug concentration and to predict concentrations in individual patients, PK models have been developed (152–154). Clearly, the currently available data are not sufficient to inform a practise guideline for TDM of Treosulfan in paediatric ALL—the relationship of Treosulfan exposure to leukaemia-free survival has not been described. A number of clinical trials incorporating Treosulfan PK evaluation are underway that may provide additional insights. In particular, the PK data on Treosulfan from the FORUM trial are eagerly awaited.

## Introducing Clofarabine into Conditioning Regimens

Clofarabine is a second-generation purine nucleoside analogue that was designed to improve outcomes and minimise toxicity in the treatment of acute leukaemia. It inhibits DNA synthesis and repair and also disrupts the mitochondrial membrane resulting in programmed cell death. It has been studied widely in the setting of relapsed/refractory ALL over the past decade and was approved for the use in refractory or relapsed ALL in children by the FDA in 2004.

It has an acceptable toxicity profile with more frequent adverse reactions including febrile neutropenia, nausea/anorexia, cytokine-release–like events, skin rash and hand-foot syndrome (155–157). This safety profile supports the feasibility of combining Clofarabine with other effective agents based on pharmacological properties and mechanisms of action. In particular, the combination of Clofarabine, Cyclophosphamide and etoposide for conditioning has been studied in children with relapsed or refractory ALL undergoing HSCT and has been found to be well-tolerated, with overall response rates of 28–67% (158–160).

### Use of Clofarabine in HSCT Conditioning

One advantage of Clofarabine is that it is not associated with the neurotoxicity seen with other similar nucleoside analogues. In order to reduce toxicity but sustain efficacy, studies both *in vitro* and *in vivo* have been done where nucleoside analogues replace alkylating agents. *In vitro* cell line studies showed the clear synergistic cytotoxicity of Clofarabine and Fludarabine, which was further enhanced by adding Busulfan. This finding led to the combination of Clofarabine, Fludarabine, and Busulfan being investigated by the MD Anderson group (161, 162).

In that randomised controlled trial, 51 adult patients with high-risk myeloid leukaemias were randomised to receive Clofarabine-Fludarabine-Busulfan conditioning across four treatment arms that differed with respect to the Clo and Fludarabine dosing used. Initial findings were encouraging with regard to safety and antileukemic activity (162). Longer follow up of this expanded cohort (*n* = 70) confirmed the safety, OS and PFS advantage of the arms with higher Clofarabine doses and lower Fludarabine doses (163).

The same group studied Clofarabine and Busulfan in 107 adults undergoing HSCT for ALL (164, 165). With a median follow up of 3.3 years, 2-year leukaemia-free survival was 51% (being best in CR1 patients, at 62%), and NRM was 6% at day 100 and 18% at 2 years. These outcomes compare favourably with reports of adult patients with ALL in CR1 treated with myeloablative TBI-based regimens.

There are few data published on the use of Clofarabine for HSCT conditioning in paediatric patients. A retrospective analysis in paediatric AML using a common backbone of induction chemotherapy followed by three different chemotherapy conditioning regimens suggested that Clofarabine-Fludarabine-Busulfan had good anti-leukaemic activity with low NRM. In comparison, Busulfan-Cyclophosphamide was associated with higher relapse incidence, while Busulfan-Cyclophosphamide-Melphalan was associated with higher incidence of acute GVHD (166).

In a cohort of 60 paediatric ALL patients undergoing HSCT after Clofarabine-Fludarabine-Busulfan conditioning, the 2-year estimated EFS probability was 72.0% ± 6.0, with significantly lower EFS observed in patients with MRD positivity prior to HSCT. Two-year TRM probability was low at only 5.0% ± 2.8 and no VOD was seen.

At the time of writing, there were no ongoing clinical studies of Clofarabine use in HSCT conditioning regimens.

## Optimising the Entire Conditioning Regimen

We have tried to address the issues around optimising the PK and PD of the individual alkylators in the conditioning regimen, but it is equally important to address the impact of the entire package on efficacy and toxicity.

### Substituting Alkylating Agents

For Busulfan, acute and chronic toxicities remain a matter of concern even when Busulfan target exposures are strictly controlled (50, 167). As shown by several studies, the use of multiple alkylating agents in conditioning regimens is a predictor of acute toxicity in paediatric patients (36, 37). For this reason and based on adult experience, the nucleoside analogue Fludarabine—an inhibitor of DNA, RNA and protein synthesis—has been introduced as an immunosuppressive agent in the replacement of Cyclophosphamide in paediatric transplantation. The majority of data comparing Fludarabine-Busulfan to Busulfan-Cyclophosphamide conditioning regimens come from adult patients, although some of these studies included children and adolescents. The meta-analysis by Ben-Barouch et al. included studies with paediatric ALL patients (168). The authors reported that a lower risk of NRM was associated with Fludarabine-Busulfan vs. Busulfan-Cyclophosphamide, while OS was similar between the two regimens. The same study found that Fludarabine-Busulfan was associated with lower risk of SOS than Busulfan-Cyclophosphamide. However, when only considering randomised controlled trials, the SOS risk was similar between the two regimens. A higher risk of microbiological infections was associated with the Busulfan-Cyclophosphamide regimen. Other assessed outcomes (GvHD, relapse, engraftment and mucositis) were similar between the two regimens. The meta-analysis concluded that Fludarabine-Busulfan and Busulfan-Cyclophosphamide regimens have similar efficacy, but Fludarabine-Busulfan regimens are slightly more favourable in terms of toxicity profile.

Two important studies have compared Busulfan-Cyclophosphamide and Fludarabine-Busulfan regimens in paediatric HSCT. In the first, Bartelink et al. compared the data of patients prospectively recruited 64 patients (9 ALL) who received Fludarabine-Busulfan conditioning with retrospective data of 50 (5 ALL) patients who received Busulfan-Cyclophosphamide. ALL patients received melphalan (Mel) in addition to Busulfan-Cyclophosphamide. Much like the picture in adults, EFS and OS were similar between conditioning groups, while the risk of TRT such as SOS, chronic GvHD, acute lung toxicity and viral reactivations were lower in patients who received Fludarabine-Busulfan (169). Rates of acute GvHD were similar between the two groups. As shown by more recent data, the use of three alkylating agents is correlated with the occurrence of acute toxicity compared to patients with two or one alkylating agent (36). Mel-containing conditioning regimens were also associated with acute toxicity risk (37). The use of Mel could therefore have contributed to the observed higher toxicity in Busulfan-Cyclophosphamide-Melphalan group in the study by Bartelink et al. A sub-analysis of that study that excluded ALL patients (for whom Mel was indicated), showed less toxicity in patients receiving Fludarabine-Busulfan compared with Busulfan-Cyclophosphamide. The comparison between outcomes of ALL patients receiving Busulfan-Cyclophosphamide-Melphalan vs. Fludarabine-Busulfan was not reported by the authors (169). The second study, by Harris et al., compared Fludarabine-Busulfan and Busulfan-Cyclophosphamide using retrospective data from 1,781 transplanted children. Post-relapse survival was inferior in patients receiving Fludarabine-Busulfan vs. Busulfan-Cyclophosphamide, leading to an inferior OS in those patients (170). In contrast to the Bartelink et al. study, this study showed no difference in transplant-related toxicity and TRM between conditioning groups (170). This suggests that one may still consider the addition of a third agent, but on the backbone of Busulfan-Fludarabine rather than Busulfan-Cyclophosphamide.

In contrast to Busulfan-Cyclophosphamide, there is evidence of a PK drug–drug interaction between Busulfan and Fludarabine. Two studies have shown a significantly decreased clearance of Busulfan when co-administered with Fludarabine (82, 171). As the effect sizes related to Busulfan co-administration reported in these studies were fairly small, the clinical significance of this interaction is likely to be minimal. As Busulfan has a narrow therapeutic window, even this small effect size should be considered for accurate dose individualisation of Busulfan. Furthermore, Busulfan-related toxicities in patients co-administered Busulfan and Fludarabine are also exposure dependent. A higher inter-dose variability was reported in patients receiving a Fludarabine co-administered with Busulfan, than that observed with Busulfan-Cyclophosphamide (172). TDM is therefore important to control for this increased PK variability observed when Busulfan is used alongside Fludarabine in conditioning regimens.

### Pharmacokinetics of Fludarabine

There is a small but emerging literature on Fludarabine PK in Paediatric Transplantation. Retrospective data suggested high levels were associated with more toxicity, particularly in the setting of renal impairment (173). A more recent prospective multicentre study again showed that renal impairment predictably increased AUC. In this paediatric study, it is likely that many of the patients had reduced intensity grafts, some received fludarabine alone and so the low TRM made it difficult to demonstrate if there was an relationship between exposure and TRM (174). Another paediatric study also found no association between exposure and clinically important end-points (175).

More interestingly, there has been a first attempt to look at the impact of the pharmacokinetics of Fludarabine in combination with Busulfan (176). Rather than a multivariate analysis of the impact of the PK of both Fludarabine and Busulfan independently and then looking for any interaction, the paper describes the impact of Fludarabine PK within a retrospective cohort of patients who were all given a set dose of 160 mg/m^2^ of Fludarabine combined with what is described as a targeted dose exposure of Busulfan. In fact, although an AUC of 90 mg.h/L was targeted, the mean exposure to Buslfan achieved was 96.1, with a wide range of AUC from 59 to 120 mg.h/L. Within this large series of adult and paediatric patients, including some leukaemias, and with a consequent much higher rate of TRM (28%) than the purely paediatric studies quoted above, the authors found that higher exposure associated with more toxicity and lower levels associated with more rejections. They suggested that an optimal cumulative exposure could be targeted by refinement of the current surface area based dosing, or measured as part of a TDM strategy. Give the variability in the exposure to Busulfan, which was not explored in this retrospective study, this is an illustration of the way forward.

### Pharmacokinetics of the “Serotherapy”

The chemotherapy drugs used in transplant conditioning are not given in isolation. Additional immunosuppression, depending on donor type and cell source, is added in, typically in the form of agents such as Anti-T cell polyclonal antibodies or monoclonal antibodies, such as Alemtuzumab. Their use is considered in a separate chapter of this issue.

### Adding to Busulfan

The Busulfan-based protocol used in the FORUM study added Thiotepa (10 mg/kg divided into two doses) to the Busulfan and Fludarabine. This combination is based upon protocols mainly studied in adult patients (177, 178), umbilical cord blood transplantation (179–181), haploidentical HSCT (180, 182), and reduced intensity regimens (183). The rationale behind the addition of Thiotepa was to improve the engraftment rates in adult umbilical cord blood transplanted patients, which was insufficient under a Fludarabine-Busulfan regimen (179, 184, 185). The original protocols used only 3 days of Busulfan at 3.2 mg/mL daily, thus a lower cumulative dose than myeloablative regimens. In FORUM, this protocol was used as the Busulfan-based conditioning arm but with the standard 4 days of conditioning and myeloablative target exposures suggested. In adult AML, intensifying Fludarabine-Busulfan-Thiotepa conditioning with full myeloablative doses of Busulfan resulted in significantly lower relapse [hazard ratio (HR) 0.47; *p* = 0.005] but higher NRM (HR 2.69; *p* < 0.001) compared with a myeloablative Fludarabine-Busulfan regimen (178). Leukaemia-free survival and OS was similar between the two regimens. Fludarabine-Busulfan-Thiotepa has been reported also to result in a lower relapse rate (HR 0.6; *p* = 0.02) and similar OS compared with Busulfan-Cyclophosphamide in adult AML patients (177, 178). Fludarabine-Busulfan-Thiotepa had not been studied in the conventional matched donor setting in ALL paediatric patients prior to the FORUM study. It remains unknown if this combination results in optimal outcomes in paediatric ALL and should be tested against other Busulfan-based regimens in paediatric ALL patients is therefore needed.

### Adding to Treosulfan

As described in Section Optimizing the Use of Treosulfan above, the favourable toxicity profile of Treosulfan, combined with its limited activity when combined with Fludarabine alone, led to the addition of a third agent, often Thiotepa or Melphalan.

### Pharmacokinetics of the Whole Conditioning Regimen

When using potentially toxic drugs at high doses for a short period of time, after gaining as much PK and PD information as possible from investigations of each single drug, it becomes important to look at the impact of the agents in combination. For ALL, we have added Thiotepa to Fludarabine partnered with Busulfan or Treosulfan, or used Clofarabine. We then have to consider the impact of the serotherapy used. It is naïve to believe that the complex relationship between disease and disease status, type of donor and cell source used after giving multi-agent chemotherapy combined with serotherapy will have a simple relationship to even complex descriptors of any one of the conditioning agents used. For the next phase of our international PK/PD effort, we should attempt to share data to integrate information regarding each element of the conditioning. In this way, we can move closer to our goal of optimising conditioning for each individual patient.

## Conclusion: Where Do We Go From Here?

Although only initial results are available from the FORUM trial (34), these give us some clear insights that can help to determine where we should go to from here:

- TBI was superior to both Treosulfan-based and Busulfan-based chemo-conditioning.
- This superiority extended across all sub-group analyses, regardless of age, phenotype, MRD status, donor type, remission status, timing, and type of relapse.
- TRM was higher in the chemo-conditioning arms compared with the TBI arm (*p* = 0.027) and tended to be higher with Treosulfan-based vs. Busulfan-based conditioning.

This clearly indicates that any attempt to non-specifically increase dosing for chemo-conditioning would result in a similar, dismal outcome to that observed 20 years ago in the PBMTC Study (20).

In addition to HSCT following TBI-based conditioning being effective therapy for those over 4 years of age with ALL (whether or not they have precursor B-cell lymphoblastic leukaemia), alternative therapies including chimeric antigen receptor (CAR) T-cell therapy have become available. Whether the availability of CAR-T cells will influence the choice of a chemotherapy-based vs. TBI-based conditioning is outside the scope of this review.

For patients under 4 years of age (or indeed potentially those under 3 years of age—a subject of debate) requiring HSCT, the life-long adverse effects of irradiation will drive the majority of paediatric transplanters to persist in optimising and using chemo-conditioning. Therefore, going forward, this is the group where we need to refine chemo-conditioning regimens. Although the three-drug combination of Busulfan-Fludarabine-Thiotepa has been used in significant numbers of patients, it worth noting that most of these patients were not paediatric patients with ALL and did not receive a matched donor graft (177, 179, 182, 183, 186). Furthermore, the dosing of Busulfan used in these published studies was three-quarters of the standard dose and we have not finished analysing the impact of Busulfan dose in the context of the FORUM study. This work will allow us to study the impact of various levels of exposure to Busulfan in children with ALL and determine whether factors such as cumulative dose given, cumulative exposure, method of dosing (such as once vs. multiple times per day) and/or pharmacogenomics will allow us to optimise individualised Busulfan dosing. Such dosing could then be carried forward into future prospective studies aiming to provide the best anti-leukaemic control with the least toxicity.

At the same time, analysis of the Treosulfan PK in the Treosulfan arm of the FORUM trial may suggest a way of optimising delivery of Treosulfan-based conditioning regimens. Particularly in the youngest patients, it is likely that TDM of Treosulfan will be indicated (149).

We also have to consider the possibility that further clinical data may emerge from new chemotherapy combinations, such as those containing Clo, that have good enough clinical outcomes to support such regimens being evaluated as one arm of future prospective studies.

Given the recent closure of randomisation to chemo-conditioning vs. TBI in the massive international effort of FORUM, it is likely to be some years before investigators are prepared to take on and/or can assemble the necessary resources to conduct another large prospective randomised study in paediatric ALL. As the number of patients <4 years old with ALL is limited, a study in this population would require a truly global effort in order to evaluate chemo-conditioning and could perhaps be conducted as part of an expanded “Interfant” collaborative protocol. Even with a global effort, numbers will mean a non-randomised study is more feasible, but can be based around further analysis of the detailed results of the Busulfan and Treosulfan arms of the FORUM trial and design an optimised chemotherapy-based alternative to TBI for conditioning.

## Author Contributions

All authors listed have made a substantial, direct, and intellectual contribution to the work and approved it for publication.

## Funding

KB and MA were supported by the Cansearch Foundation.

## Conflict of Interest

The authors declare that the research was conducted in the absence of any commercial or financial relationships that could be construed as a potential conflict of interest. The handling editor declared a shared consortium with the authors at time of review.

## Publisher's Note

All claims expressed in this article are solely those of the authors and do not necessarily represent those of their affiliated organizations, or those of the publisher, the editors and the reviewers. Any product that may be evaluated in this article, or claim that may be made by its manufacturer, is not guaranteed or endorsed by the publisher.
